# Efficiency and safety evaluation of prophylaxes for venous thrombosis after gynecological surgery

**DOI:** 10.1097/MD.0000000000020928

**Published:** 2020-06-19

**Authors:** Ruidi Yu, Faridah Nansubuga, Jun Yang, Wencheng Ding, Kezhen Li, Danhui Weng, Peng Wu, Gang Chen, Ding Ma, Juncheng Wei

**Affiliations:** a Department of Obstetrics and Gynecology; b Division of Vascular Surgery, Hepatic Surgery Center, Department of Surgery, Tongji Hospital, Tongji Medical College, Huazhong University of Science and Technology, Wuhan, People's Republic of China.

**Keywords:** antithrombin, gynecological surgical procedures, heparin, low-molecular weight, risk factors, venous thrombosis

## Abstract

**Background:**

In this study, we investigate the incidence of venous thrombosis (VT), and evaluate the effectiveness and safety of 3 major thromboprophylaxes and the potential risk factors for VT in women undergoing surgery for a gynecological malignancy.

**Methods:**

We performed a randomized controlled trial of 307 patients undergoing laparoscopic surgery for gynecological malignancies at a single institution from January 2016 to October 2017. Patients were divided into 3 groups: one receiving a half dose of low-molecular-weight heparin sodium injection (FLUXUM, Alfa Wassermann, Italy) delivered by injection, one receiving a full dose of FLUXUM, and a third group receiving an Argatroban injection.

**Results:**

None of the patients in our study developed a pulmonary embolism, bleeding, or infectious complications. There were no statistical differences in the rate of deep venous thrombosis (DVT) (0%, 0%, and 2.38%) and the superficial venous thromboembolism (SVT) (15.66%, 8.97%, and 18.6%) among the 3 groups. None of the patients developed symptomatic VT. The effect of treatment on alanine aminotransferase and aspartate aminotransferase differed between the groups, with a minimal effect in the Argatroban group, and all 3 methods resulted in minimal impairment of renal function. Decreased hemoglobin, elevated levels of D-dimer, and prothrombin time were closely related to thrombogenesis.

**Conclusion:**

In conclusion, the incidence of postoperative thrombosis in gynecological malignancy among these Chinese people is not as low as we had originally presumed. Argatroban is not more effective than Parnaparin as a direct thrombin inhibitor, but it has less influence on liver function, which is beneficial for patients undergoing chemotherapy. Hemoglobin, D-dimer, and prothrombin time may be used to predict or detect thrombogenesis.

## Abstract 2

Supplemental Digital Content is available in the text

## 1 Introduction

Venous thrombosis (VT) includes deep venous thrombosis (DVT) and superficial venous thrombosis (SVT). Lower-limb SVT shares the same risk factors as DVT, and it can propagate into the deep veins.^[1]^ Patients with SVT have a nearly 14% risk of venous thromboembolism (VTE) over 10 years, with a 3.3% risk of VTE in the 3 months following the SVT.^[2,3]^ VTE is composed of DVT and pulmonary embolism (PE). DVT occurs most frequently in the legs.^[4]^ Patients undergoing surgeries for gynecological malignancies are considered to be at high risk of thrombosis, due to advanced age, cancer diagnosis, pelvic mass compressing the major pelvic veins, endothelial cell injury during pelvic lymph node dissection, lengthy surgical procedures, and thrombogenic chemotherapy.^[5–9]^ PE following DVT accounts for 40% of deaths after gynecological operations.^[10]^ With prophylaxes, VTE rate of patients after gynecological surgery is from 2.7% to 4%.^[11,12]^ Most DVTs after gynecological surgeries do not have typical symptoms.^[13–15]^ Death might occur within 30 minutes of the onset of PE; this time is inadequate for any therapeutic intervention after symptoms appear.^[16–19]^ Therefore, accurate diagnosis is a challenge. The application of appropriate thromboprophylaxis, including unfractionated heparin, or low molecular weight heparin (LMWH), which are commonly used and widely accepted, has been reported as efficacious.^[20–22]^ Existing guidelines for perioperative thromboprophylaxes are listed in the review by Cantrell et al.^[23]^

Argatroban is a direct thrombin inhibitor. In contrast to heparins that require antithrombin to inhibit thrombin, Argatroban binds to thrombin independent of antithrombin, inhibiting both plasma- and fibrin-bound thrombin.^[24–29]^ It is an alternative to heparin, which has a risk of inducing thrombocytopenia, and has less influence on renal function than LMWH.^[30–33]^ However, few clinical studies have been published regarding the effectiveness and safety of the early initiation of Argatroban therapy in the management of perioperative VT in patients undergoing gynecological surgery.

Although VT events have been well documented globally and it has been widely accepted for many years that the incidence of VT is lower in Chinese people than in Caucasians, there is no conclusive data or evidence.^[34–36]^ Therefore, the specific rate of thrombosis in the Chinese population remains to be determined. Thus, in this study, we investigate the incidence of VT and evaluate the effectiveness and safety of 3 major thromboprophylaxes and the potential risk factors for VT in women undergoing surgery for a gynecological malignancy.

## 2 Methods

### 2.1 Study design

Our goal is to evaluate the effectiveness and safety of low molecular weight heparin sodium injection (FLUXUM) and Argatroban, and investigate the rate of perioperative thrombosis after gynecological surgery. We hypothesized that Arg is more effective and safer than FLUXUM, and the rate of perioperative thrombosis is similar to previous research.^[34–38]^ We performed a prospective randomized controlled trial of patients undergoing laparoscopic surgery for a gynecological malignancy at a single institution from January 2016 to October 2017, with a 3-month follow-up period. Doctors from the Department of Ultrasound and the clinical laboratory were all blinded to patient grouping. The trial was registered on ClinicalTrials.gov (NCT02935530).

### 2.2 Ethics

Our research was approved by the institutional Review Board of Tongji Hospital at the Tongji Medical College of the Huazhong University of Science and Technology (Wuhan, China) and written informed consent was obtained from each patient before treatment.

### 2.3 Participants

A total of 315 patients, who underwent laparoscopic gynecological surgery in Tongji Hospital, one of the largest general hospitals in Hubei province from January 2016 to October 2017, were accepted into the study. Inclusion criteria included the following: Chinese women from Hubei province; a diagnosis of ovarian, cervical, or endometrial cancer; and undergoing curative surgery. Patients over 70, with symptomatic preoperative VTE or with an abnormality of their liver and renal function (more than 3 times the normal reference value) were excluded from the study. Patients were also excluded if the preoperative platelet count was ≤ 75 × 10^9^, if they were taking anticoagulants, if they had intraoperative vascular injuries, or if they were concurrently participating in other clinical trials.

### 2.4 Interventions

Patients were numbered from 1 to 315. Using a random number table, the patients were randomly assigned to 3 intervention groups. The first group was given half the usual prophylactic dose, 2125 I.U. anti-factor Xa per day, of LMWH (FLUXUM, Alfa Wassermann, Italy), by subcutaneous injection (Half-FLU group). The second group was given the full prophylactic dose, 4250 I.U. anti-factor Xa per day, by subcutaneous injection (FLU group).^[39]^ The doses used to prevent clots were given according to the drug instruction by Alfa Wassermann, regardless of body weight, and half dose in the instruction was suggested to be used in patients who have high risk of bleeding. The third group was given 20 mg Argatroban IV (Tianjin Pharmaceutical Research Institute Co, LTD, **Tianjin**, China), for 3 hours per day (Arg group).^[24,40]^ Graduated compression stockings were used routinely.^[41–43]^ Patients received the first doses of prophylaxis 6 hours after surgery, followed by a daily dose for 28 days.^[41,43]^ We monitored the coagulation function of patients throughout the trial.

### 2.5 Outcomes

Information was obtained from the medical records of the patients. The following tests were performed: routine blood test, routine urine analysis, coagulation function, and blood biochemistry prior to surgery and on postoperative day 1 (POD1), 7 (POD7), 30 (POD30), 60 (POD60), and 90 (POD90). A clinical diagnosis of thrombosis was confirmed on complete compression ultrasonography (CUS); this was suggested as a screening modality for VT in high-risk perioperative patients, as it has a high sensitivity and specificity.^[4,44–46]^ CUS of the arteriovenous system of the lower limbs was performed on POD7, 30, 60, and 90. All the patients were examined and monitored for any signs of VTE, bleeding, or infection.

### 2.6 Statistical analysis

We used the SAS (Version 9.4) software package (SAS Institute, Inc, Cary, NC) for statistical analysis. Logistic regression was used to compare the rates among the groups. We used a *t* test to compare 2 groups whose data satisfied the normal distribution, and analysis of variance to compare the 3 groups. The mean result (95% confidence interval) was used. We used Mann–Whitney *U* test and a median (interquartile range) for data not satisfying the normal distribution. Differences were considered to be statistically significant when *P* < .05. We used the Pass 15 software package to calculate the sample size, taking the predicted SVT proportions of the 3 groups as 0.5, and worked out that no less than 100 per group was enough.^[47,48]^

## 3 Results

### 3.1 Proportion of postoperative VT

**Figure 1:**
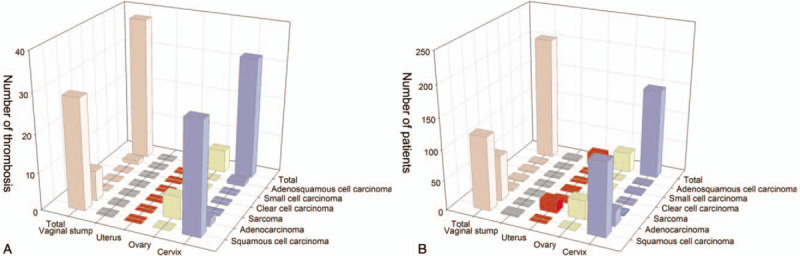
A, Number of patients with venous thrombosis following gynecological surgery in relation to tumor histopathology and site. B, Number of patients with venous thrombosis with different pathologic types of tumor and sites. They were not statistically significantly different.

Between January 2016 and October 2017, 307 patients who underwent surgery for the management of an invasive gynecological cancer at Tongji Hospital were followed up (Table 1). Over the 3-month follow-up, 245 valid data sets were collected, as 62 patients did not complete the color Doppler ultrasonography, and none of them presented symptomatic events. Thirty-six of the 245 patients had a thrombosis (14.69%). Of these, 13 had been given a half-FLU (83 total patients in this group, thrombosis rate 15.66%), 7 FLU (78 total patients in this group, thrombosis rate 8.97%), and 16 had been given Arg (86 total patients in this group, thrombosis rate 18.6%). No statistically significant differences were found in the rates of thrombosis among the 3 groups. Only 2 patients in the Arg group developed DVT (2.38%) (Table 2, Fig. 1).

**Table 1:**
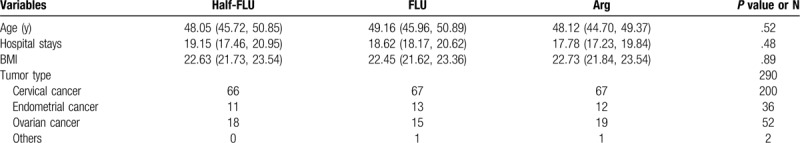
The demographic and patient characters in each group.

**Table 2:**
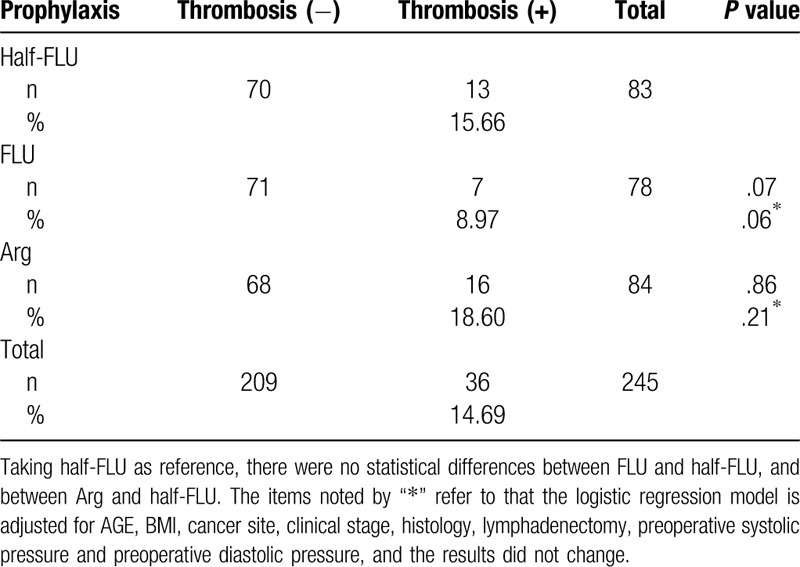
The rate of perioperative thrombosis in separate groups and the comparison between them.

Posterior tibial VT was detected in 2 patients. These were detected on POD7 in the right leg of patients in the Arg group, one of whom had cervical cancer, and the other ovarian cancer. We changed the antithrombotic regime to a full dose of Parnaparin after the thrombi were found. Among the 3 groups, there were no significant differences in the rates of DVT (*P* = .37), and in the half-FLU (0%), and FLU group (0%), and 2 patients in Arg group (2.38%) developed DVT. Great saphenous vein thrombosis occurred in only 1 patient who was given Arg as thromboprophylaxis and diagnosed as having an ovarian borderline micro papillary serous tumor. The patient had a muscular VT in the left calf, which was detected on POD7. She was discharged 2 days later without oral anticoagulants or other antithrombotic drugs. A great saphenous VT in the left calf and a muscular VT in the right calf were found on POD30 and POD60, respectively. The other 33 patients each had a muscular VT in the calf, with 29 of them being unilateral (18 left, 11 right), 3 of which were detected on POD30, and the other 26 on POD7. The 4 who had bilateral thrombi, were detected on POD7. Two of the 245 patients had DVT, and none of them had PE or other related symptoms. There was no hemorrhage related to anticoagulant use (Supplementary Table 1). In the 13 patients in the Half-FLU group with a thrombosis, this was detected by color Doppler ultrasonography on POD7. In 5 of the 7 patients in the FLU group with a thrombosis, this was detected on POD7, while in the other 2 patients, it was detected on POD30. Similarly, in the 14 patients in the Arg group with a thrombosis, it was detected on POD7, and in the remaining 2 of the 16 patients with a thrombosis in the Arg group, thrombosis were detected on POD30 (Table 3).

**Table 3:**
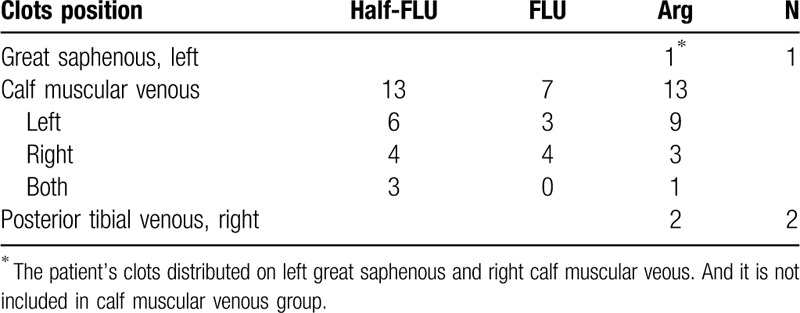
The position of the clots when first detected.

### 3.2 The effects of different forms of thromboprophylaxes on renal and liver function

To study the side effects of these 3 anticoagulant therapies on liver and renal function, we compared the levels of alanine aminotransferase (ALT), aspartate aminotransferase (AST), total bilirubin (TBil), alkaline phosphatase (ALP), γ-glutamyl transpeptidase (γ-GTP), lactic dehydrogenase (LDH), blood urea nitrogen (BUN) creatinine (Cr), and uric acid (UA) on POD7, POD30, POD60, and POD90 between the groups. The anticoagulants were given on the first day after surgery. Laboratory test results on the first day after surgery (before using anticoagulants) were used as the base line (Supplementary table 2).

The levels of ALT, AST, and γ-GTP showed significant differences on POD7 across the 3 groups. The levels of ALT were 14.0, 24.5, and 12.0 U/L, respectively, in the half-FLU, FLU, and Arg groups (*P* = .000), and the levels of AST were 19.5, 27.0, and 16.0 U/L, respectively (*P* = .000). In addition, the levels of γ-GTP were 29.0, 33.0, and 21.0 U/L in the half-FLU, FLU, and Arg groups, respectively (*P* = .01) (Supplementary table 2a). We further explored where the differences came from (Supplementary table 2b), finding significant differences in ALT between the FLU and Arg groups (*P* < .0001). There were significant differences in AST between the Half-FLU and FLU groups (*P* = .0086), and between the FLU and Arg groups (*P* < .0001), and borderline significant differences between the Half-FLU and Arg groups (*P* = .02). The difference in POD7 γ-GTP between the FLU and Arg groups was significant (*P* = .0081), but the differences between the Half-FLU and FLU groups, as well as between the Half-FLU and Arg groups, were borderline significant. Thus, FLU had the greatest degree of liver damage, and this was less in patients using Arg. There were no significant differences in TBil, LDH, and BUN among the groups throughout the duration of the study. On POD90, ALP levels across the groups were significantly different, but the ALP levels did not rise relative to the former levels. Therefore, we could not conclude that the anticoagulants influenced ALP levels.

**Figure 2:**
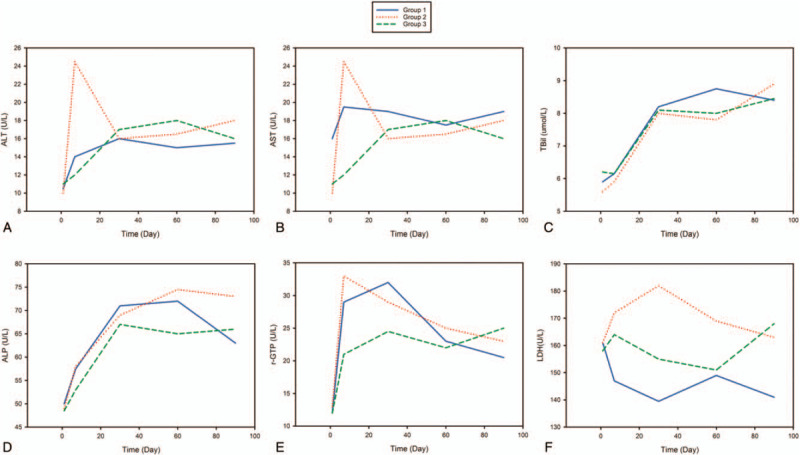
Blood tests for renal and liver function in different treatment groups during 90-day follow-up. A, The level of ALT in each group (*P* < .0001 on POD7). B, The level of AST in each group (*P* < .0001 on POD7). C, The level of TBil in each group. D, The level of ALP in each group. E, The level of γ-GTP in each group (*P* = .0149 on POD7). F, Level of LDH in 3 treatment groups. No significant differences were observed. The first group (Half-FLU) was given half the usual dose of low molecular weight heparin sodium (FLUXUM) 2125KU subcutaneous injection, the second group (FLU) was given a full usual dose of FLUXUM 4250KU subcutaneous injection, and the third group (Arg) was given an Argatroban 20 mg injection. γ-GTP = γ-glutamyl transpeptidase, ALP = alkaline phosphatase, ALT = glutamic-pyruvic transaminase, Arg = argatroban IV injection, AST = glutami-oxalacetic transaminase, FLU = full usual prophylactic dose of low molecular weight heparin sodium injection, FLUXUM = low molecular weight heparin sodium injection, Half-FLU = half usual prophylactic dose of low molecular weight heparin sodium injection, LDH = lactic dehydrogenase, TBil = total bilirubin.

**Figure 3:**
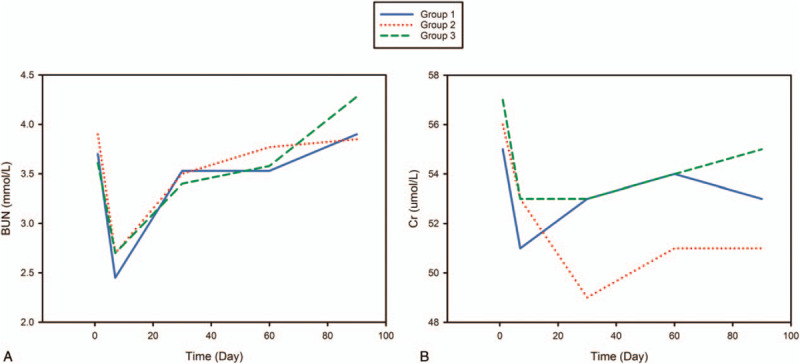
BUN and Cr, levels in different groups during the 90-day follow-up. A, BUN levels in each group. B, Cr levels in each group. BUN = blood urea nitrogen, Cr = creatinine.

To know whether and when the levels of the blood parameters above changed, we also compared these on POD7, POD30, POD60, and POD90 with these on the first day after the operation (POD1) separately in the different groups (Supplementary Table 3, Figs. 2 and 3). In the Half-FLU and FLU groups, ALT, AST, and ALP had changed significantly since POD7, and did not return to their former level by POD90. In the Arg group, there was no significant difference between POD7 and POD1; thus, we concluded that these items changed later, but significant differences were found from POD30 to POD90. The level of TBil in all 3 groups was significantly higher from POD30 to POD90 than on POD1, and the level of γ-GTP increased significantly from POD7 to POD90. In the Half-FLU group, LDH, BUN, and Cr were statistically different on POD7 compared with POD1, but not at later times in the study. In the FLU and Arg groups, LDH had not changed, and BUN changed only on POD7. The level of Cr was statistically different from POD7 to POD90 when compared with POD1 in the FLU group, and from POD7 to POD30 in the Arg group (Supplementary Table 3, Figs. 2 and 3).

As to Cr and BUN levels, there are no significant differences among 3 groups. We could see that BUN and Cr levels in all the groups decreased provisionally. BUN and Cr are the metabolites of protein and creatine. The downward trend might result from low protein intake and limited activity after the surgery, leading to a low metabolic rate in muscle.UA levels among the groups were not significantly different. This might be because of the limited influence of the 3 forms of thromboprophylaxis on purine metabolism (Supplementary Table 2).

In elder women aged more than 50 years old, Arg group had minimal influence on liver function (ALT, AST, and γ-GTP) compared with other 2 groups (Supplementary Table 12a, Row 10, 11, 14, and 23). While in younger women, FLU group had maximal effect on ALT and AST level compared with other 2 groups (Supplementary Table 12b, Row 10 and 11). And in all of the 3 groups, women older than 50 have more obvious liver injury compared with women younger than 50 (Supplementary Table 13a, Row 4, 13, 14, 22, and 23; Supplementary Table 13b, Row 4, 13, and 30; Supplementary Table 13c, Row 3 and 4). Moreover, these were transient liver damage and there are no differences on POD90 (Supplementary Table 12 and Supplementary Table 13, Row 37–42). And none of the 3 groups had obvious injury to renal function, regardless of age (Supplementary Table 12 and Supplementary Table 13, Row 7–9, 16–18, 25–27, 34–36 and 43–45).

### 3.3 Relevant factors for perioperative venous thrombosis

Because 32 of 36 thrombosis-positive patients were diagnosed within 1 week of their operation, we used these patients as the sample group to analyze relevant factors. In a univariate analysis of the demographic characteristics of the patients, those who had a thrombosis were significantly older than those who did not (51.84(48.84, 54.85) years vs 48.07 (46.86, 49.28) years *P* = .03) (Table 4, Supplementary table 4, Supplementary Table 5). In the Chinese population, the average age at menopause is about 50 years. We thus used this age as the cut-off value. The rate of thrombosis was much higher in those older than 50 years (Supplementary Table 6). Thrombosis-positive patients also had longer hospital stays (*P* = .05). However, no difference was noted based on body mass index (BMI) and preoperative blood pressure (systolic pressure, *P* = .69; diastolic pressure, *P* = .82). Furthermore, no difference was noted among those who had early stage cancer and those who did not. There was also no difference between patients who had undergone lymphadenectomy and those who had not. Since most patients with invasive cancer underwent lymphadenectomy, and only the minority of patients did not, their data were too limited to be compared.

**Table 4:**
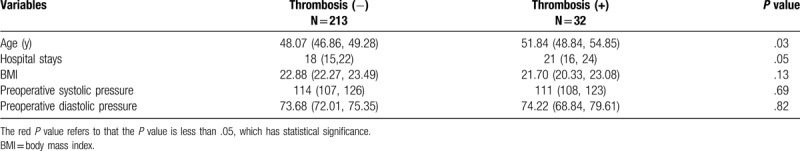
Univariate analysis of demographic, preoperative, and intraoperative characteristics of patients who had and did not have thrombosis.

With respect to postoperative diagnosis and histological type, among the 204 patients whose records were included (Supplementary Table 7), no differences were noted based on the site of the cancer diagnosis and the histological type (*P* = .08, *P* = .38, respectively) (Supplementary Table 6, Fig. 1).

### 3.4 Laboratory test results to predict and detect perioperative venous thrombosis

We investigated several laboratory tests, including total cholesterol (TC), triglyceride (TG), hemoglobin (Hb), platelet count (PLT), prothrombin time (PT), fibrinogen (FIB), activated partial thromboplastin time (APTT), D-dimer (D-D), and uric acid (UA) at different times after the surgery. According to whether the patient had a postoperative thrombosis or not, we divided the patients into 2 groups: a thrombosis (+) group and a thrombosis (−) group (Table 5, Supplementary Table 8, Supplementary Table 9, Supplementary Table 10 and 11). At initial assessment, the average preoperative hemoglobin on POD1 and POD7 were 113 and 99.83 g/L versus 119 and 106.39 g/L in the thrombosis (+) and the thrombosis (−) groups, respectively (*P* = .02, *P* = .02) (Table 5). It appears that the low hemoglobin level might be related to perioperative thrombogenesis.

**Table 5:**
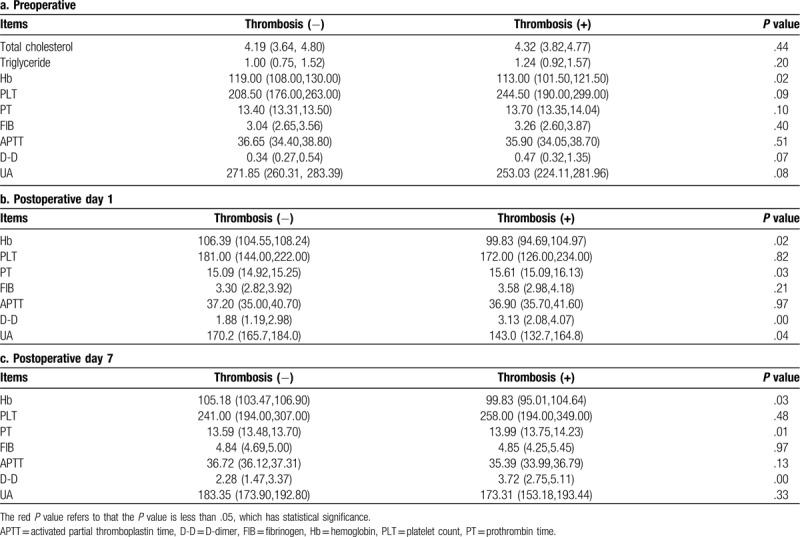
Analysis of the relationship between laboratory test results and perioperative thrombosis.

The levels of PT were 15.09 versus 15.61 seconds on POD1 (*P* = .03) and 13.59 versus 13.99 seconds on POD7 (*P* = .01) in the thrombosis (−) and (+) groups, respectively. The level of PT in the thrombosis (+) group was longer than in the (−) group. Prolonged PT might be related to perioperative thrombogenesis.

On POD1 and POD7, the D-D of both groups were significantly different (1.88 vs 3.13, *P* = .001 and 2.28 vs 3.72, *P* = .006). The level of D-D in the thrombosis (+) group was always higher than in the thrombosis (−) group during the postoperative period. Thus, a high level of D-D might signal a high risk of postoperative thrombosis. Thereafter, we further evaluated the levels of D-D on POD1 and POD7. Taking 0.5, 1.5, and 3.0 as limits, only 1.5 and 3.0 showed significant differences (Supplementary Table 7). Early elevation of D-D is related to perioperative thrombosis. A D-D higher than 1.5 should result in additional monitoring.

On POD1, the level of UA in the thrombosis (+) group was lower than in the (−) group (*P* = .04). In addition, there were no significant differences between the thrombosis (+) and (−) groups at other stages.

The levels of preoperative TC, TG, PLT, FIB, and APTT were not statistically significantly different, which means that these tests might not be useful in predicting or diagnosing perioperative thrombogenesis (Supplementary Figure 1).

## 4 Discussion

In our study, 88.89% of VT was detected within 7 days of surgery, and 100% was detected within 30 days. In a controlled trial, the incidence of DVT in gynecological cancer patients without thromboprophylaxis was 18%, and the rate of pulmonary embolism among those patients was 2.6%.^[49]^ In previous research, 38% of gynecological cancer patients developed perioperative VTE, which is much higher than that of patients undergoing benign gynecological surgery (14%).^[50]^ With the routine use of prophylaxis (thigh-high sequential compression devices), the risk of thromboembolism has been reduced to 4.2% to 6.4%,^[47,48]^ and the symptomatic VTE rate is even lower in women who undergo laparoscopic surgery for endometrial cancer.^[51,52]^ In our study, the SVT rate was 14.69%, and the DVT rate was 0.82%, which is lower than in previous reports.^[34–37]^ Great saphenous VT occurred in only 1 patient, and posterior tibial VT was detected in 2 patients. Of the patients who had a thrombosis, 91.6% had a muscular VT in the calf. This implies that the 3 forms of thromboprophylaxis are efficient. In gynecological cancer patients, it is reported that over 75% of VT is detected more than 7 days after surgery, in accordance with our results.^[53]^ Patients receiving LMWH were found to have significantly lower rates of VTE within 30 days of surgery compared with those who did not receive it.^[54]^

None of the patients developed a fatal PE, while in previous studies the range of fatal PE was between 0.2% and 0.9%.^[50,55]^ Overall, there were no instances of significant bleeding or death in our study. The results of our study demonstrated a significant decrease in VT risk and complications in patients. A possible reason for this could be the effectiveness and safety of the 3 forms of thromboprophylaxis, when used timeously.

There was no significant difference in the efficacy of the 3 different treatments. A half-FLU did not reduce the efficacy of prophylaxis compared with the full prophylaxis dose. However, a half dose, administered according to the directions of Alfa Wassermann, reduced the risk of bleeding. We found that Arg had equal efficiency compared with half-FLU and FLU.

In view of chemotherapy-induced liver and renal toxicity, thromboprophylaxis should result in minimal harm to the liver and kidneys.^[56–59]^ In a number of previous clinical trials, LMWH given by subcutaneous injection was common therapy for treatment of VTE and had a lower risk of heparin-induced thrombocytopenia (HIT) than unfractionated heparin.^[60]^ Argatroban has been reported as a feasible alternative in patients with HIT,^[61–64]^ without increasing the risk of bleeding.^[30]^ It is approved in the US and Canada for both prophylaxis and treatment of thrombosis in patients with HIT.^[31,61,65–67]^ It has a small molecular weight, a peptidomimetic structure, reversible binding to thrombin, and a nonimmunogenic nature. It can be differentiated from other anticoagulants by its hepatic clearance, and, as a result, it does not lead to thrombocytopenia, and is not excreted by the kidneys, so has little influence on renal function.^[30–32]^ However, in our research, neither Arg nor FLUXUM had any influence on Cr or BUN, suggesting that both have minimal nephrotoxic effects. With respect to liver function, however, the degree of elevation of ALT and AST in the Arg group was less than that in the FLUXUM groups. For most patients with a gynecological malignancy, chemotherapy is necessary. For this to be possible, levels of ALT and AST, lower than 80 U/L, are required. Arg shows its superiority in this field. Moreover, half-FLU results in less live impairment than a full dose, this impairment is reversible, usually within 30 days. A mild transaminitis is common after surgery, including laparoscopic surgery, even in healthy patients.^[68]^ Thus, we cannot attribute changes in liver function to the thromboprophylaxis only. Current recommendations for Arg monitoring are to use the APTT for low doses and the activated clotting time for high doses, making monitoring easy. During the 3 months of follow-up there was no significant trend favoring FLU or half-FLU as compared with Arg.

In identifying risk factors, the Caprini score is a well-recognized strategy.^[69,70]^ It was reported that age, BMI, congestive heart failure, active malignant neoplasm, chemotherapy, previous superficial vein thrombosis, previous varicose vein procedure, chronic renal disease, neurologic disease with extremity paresis, previous central venous catheterization or transvenous pacemaker placement, trauma, any surgery, orthopedic surgery, neurosurgery, and anesthesia were potential risk factors for VT. Admission to a hospital, or nursing home, within 90 days of surgery was also a significant risk factor.^[71]^ Known risk factors for SVT include intravenous catheters, venous valvular insufficiency, pregnancy, oral contraception, infection, abuse of nicotine, history of thrombosis, obesity, malignancy and age >60 years.^[1,72,73]^ We concluded from the above that patients undergoing surgery for a gynecological malignancy were at high risk of thrombosis.^[74]^ We thus chose these variables to evaluate. Several demographic characteristics of patients were analyzed to identify any further underlying risk factors that increased the incidence of postoperative VT for patients with gynecological malignancy. These included blood pressure, BMI, age, characteristics of the tumor, histology subtype, and the site of the cancer. However, in our study, only age showed a close association to the incidence of VT, confirming the findings of other authors.^[71,74]^ From our findings, BMI does not appear to have significant influence on the risk of VT, which is not consistent with former reports. Blood pressure, clinical stage, cancer site, and histological subtype also do not influence the rate of postoperative VT. Our study appears to show a higher incidence of VT in women with ovarian compared with those with cervical cancer. However, because of limited data for ovarian cancer, endometrial, and other cancers, no statistical differences were found in our research. These results are partially consistent with the data published by Kodama et al.^[75]^ Vulvar cancer is also regarded as high risk in some studies, accompanied by ovarian cancer.^[5]^ This might be related to the invasion of the tumor and the surgery.

D-D, as a most frequently used marker for coagulation and fibrinolysis, has a high sensitivity of PE but is not specific enough.^[14,15]^ It is also an independent risk factor for VTE in patients undergoing urologic tumor surgery.^[76]^ Prior research reports that elevated leukocyte, platelet count, decreased hemoglobin, elevated levels of D-D, prothrombin fragment 1 + 2, soluble P-selectin, and clotting factor VIII are useful in risk prediction.^[77]^ In our analysis, decreased hemoglobin, elevated levels of D-D and PT are significantly important in predicting and detecting VT, consistent with the conclusions of former research.^[77–79]^ The longer PT in thrombosis (+) patients compared with the (−) group might be related to the consumption of coagulation factors.^[80]^ There was an increased incidence of VTE in patients with decreased hemoglobin or those who did not use iron supplementation. Whether administration of iron could indeed reduce VTE in cancer patients must be addressed in appropriately designed clinical trials.^[77,81,82]^ High platelet numbers have been reported as a risk factor for VTE, although the mechanism is not clear.^[81,83–86]^ Our study did not show this to be the case. Elevated TC, TG, fibrinogen, and short APTT were not predictors of postoperative thrombogenesis.

Elevated serum UA concentration has been shown in some studies, to be associated with a higher risk of thrombosis;^[87,88]^ however, this was not the case in our study, and it can thus not reliably be used as an indicator of perioperative thrombosis. This difference may be due to limited data or nutritional deficiency and hyper metabolism after operation.

In conclusion, most thrombosis is detected within 7 days of the surgery (Supplementary Table 1). Decreased hemoglobin, elevated levels of D-D and PT are useful indicators for the prediction or detection of thrombogenesis (Table 5b and 5c). Arg does not have better efficacy than FLUXUM as a direct thrombin inhibitor (Table 2). However, while Arg and FLUXUM both have minimal effects on renal function, Arg has less effect on liver function compared with FLUXUM (Supplementary Table 2 and Supplementary Table 3). The prophylaxes had greater effect on liver function in women aged more than 50 compared with women younger than 50 (Supplementary Table 13). While in younger women, FLU had maximal effect on liver (Supplementary Table 12b). These were transient liver damage. In addition, the prophylaxes did not have obvious injury to renal function, regardless of age (Supplementary Table 2, Supplementary Table 3, Supplementary Table 12 and Supplementary Table 13).

### 4.1 Limitations

In this study, due to the limited sample size, few number of DVT was recorded (Table 2). We did not perform routine preoperative CUS on every patient, and the SVT rate might thus be higher than recorded. Although Tongji hospital is one of the largest general hospitals, which contains the majority for gynecologic oncology, we also intended to conduct this study in multicenter in the coming days to get more data.

## 5 Addendum

RY and FN collected the data, analyzed them, wrote the article, and modified it. JY and WD made critical writing and revising the intellectual content. KL, DW, PW, and GC offered data and carried out the project. DM and JW contributed to concept and design.

## Author contributions

**Conceptualization:** Juncheng Wei, Ding Ma.

**Data curation:** Ruidi Yu, Faridah Nansubuga.

**Formal analysis:** Ruidi Yu.

**Funding acquisition:** Juncheng Wei.

**Investigation:** Faridah Nansubuga.

**Methodology:** Ruidi Yu, Jun Yang, Wencheng Ding.

**Project administration:** Jun Yang, Kezhen Li, Danhui Weng, Peng Wu, Gang Chen.

**Resources:** Wencheng Ding, Kezhen Li, Danhui Weng, Peng Wu, Gang Chen.

**Supervision:** Gang Chen, Ding Ma.

**Writing – review & editing:** Juncheng Wei.

## Supplementary Material

Supplemental Digital Content

## Supplementary Material

Supplemental Digital Content

## Supplementary Material

Supplemental Digital Content

## Supplementary Material

Supplemental Digital Content

## Supplementary Material

Supplemental Digital Content

## Supplementary Material

Supplemental Digital Content

## Supplementary Material

Supplemental Digital Content

## Supplementary Material

Supplemental Digital Content

## Supplementary Material

Supplemental Digital Content

## Supplementary Material

Supplemental Digital Content

## Supplementary Material

Supplemental Digital Content

## Supplementary Material

Supplemental Digital Content

## Supplementary Material

Supplemental Digital Content

## Supplementary Material

Supplemental Digital Content
