# Supplementary figures and images for: Efficiency and safety evaluation of prophylaxes for venous thrombosis after gynecological surgery
Source: Medicine (Baltimore). 2020 Jun 19;99(25):e20928. doi: 10.1097/MD.0000000000020928 (PMC7310966; doi:10.1097/MD.0000000000020928)

# Results

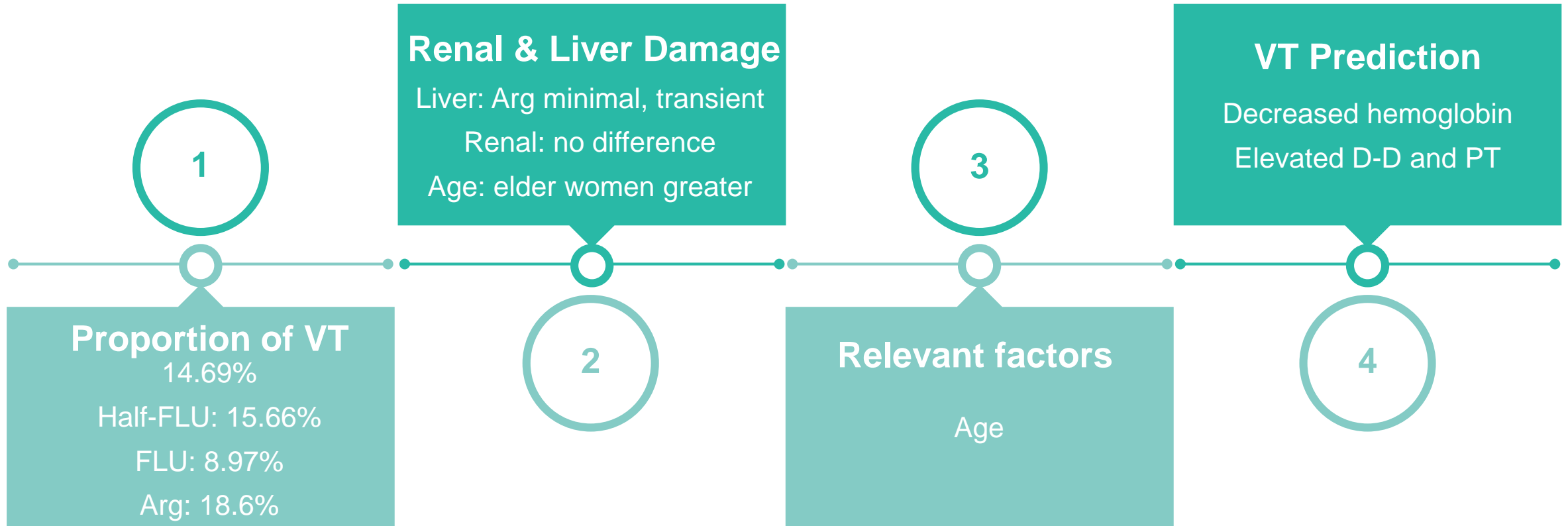

Supplement: Supplemental Digital Content [file medi-99-e20928-s001.pdf]
