# Supplementary material for: Efficiency and safety evaluation of prophylaxes for venous thrombosis after gynecological surgery
Source: Medicine (Baltimore). 2020 Jun 19;99(25):e20928. doi: 10.1097/MD.0000000000020928 (PMC7310966; doi:10.1097/MD.0000000000020928)
Supplement: Supplemental Digital Content [file medi-99-e20928-s002.docx]

**Supplementary Table 1. The incidence of the patients with thrombosis and the date of thrombosis were detected in separate groups.**

| Prophylaxis | Thrombosis n (%) | | | | |
| --- | --- | --- | --- | --- | --- |
|  | 7^th^ day | 30^th^ day | 60^th^ day | 90^th^ day | Total |
| 1.Half dose of LMWH | 13(100) | - | - | - | 13(15.66) |
| 2.Full dose of LMWH | 5(71.43) | 2(28.57) | - | - | 7(8.97) |
| 3.Argatroban | 14(87.50) | 2(12.50) | - | - | 16(18.6) |
| Total | 32(88.89) | 4(11.11) | - | - | 36(14.57) |

LMWH: Low molecular weight heparin.
