# Supplementary material for: Efficiency and safety evaluation of prophylaxes for venous thrombosis after gynecological surgery
Source: Medicine (Baltimore). 2020 Jun 19;99(25):e20928. doi: 10.1097/MD.0000000000020928 (PMC7310966; doi:10.1097/MD.0000000000020928)
Supplement: Supplemental Digital Content [file medi-99-e20928-s003.docx]

**Supplementary Table 2. The liver and renal function results in separate groups.**

**Supplementary table 2a**

| Items | Half-FLU | FLU | Arg | P-value |
| --- | --- | --- | --- | --- |
| POD1 ALT | 10.50(8.00,17.00) | 10.00(7.50,15.00) | 11.00(8.00,15.00) | .66 |
| POD1 AST | 16.00(12.00,21.00) | 17.00(13.00,21.00) | 16.00(12.00,19.00) | .62 |
| POD1 TBil | 5.90(4.50,7.80) | 5.60(4.09,8.00) | 6.20(4.50,8.77) | .33 |
| POD1 ALP | 50.00(42.00,59.00) | 49.00(43.00,59.00) | 48.50(39.75,59.00) | .69 |
| POD1 γ-GTP | 12.00(9.00,20.00) | 13.00(9.00,21.00) | 12.00(9.00,18.00) | .83 |
| POD1 LDH | 161.00(137.00,176.50) | 161.00(133.50,184.50) | 158.00(133.00,188.00) | .95 |
| POD1 BUN | 3.70(2.74,4.59) | 3.90(3.08,5.09) | 3.61(2.90,4.70) | .20 |
| POD1 Cr | 55.00(47.00,62.00) | 56.00(50.00,64.00) | 57.00(50.00,65.00) | .39 |
| POD1 UA | 161.30(123.75,210.05) | 160.00(126.00,206.50) | 174.30(130.25,211.00) | .75 |
| POD7 ALT | 14.00(9.00,32.00) | 24.50(13.00,44.25) | 12.00(9.00,19.00) | .000 |
| POD7 AST | 19.50(14.00,33.25) | 27.00(18.00,55.00) | 16.00(12.00,21.25) | .000 |
| POD7 TBil | 6.15(4.57,7.48) | 5.90(4.70,7.80) | 6.15(4.80,7.50) | .91 |
| POD7 ALP | 57.50(46.75,72.00) | 58.00(47.00,69.00) | 53.00(42.00,63.25) | .06 |
| POD7 γ-GTP | 29.00(17.00,46.75) | 33.00(16.75,60.00) | 21.00(15.50,31.00) | .01 |
| POD7 LDH | 147.00(123.50,165.50) | 172.00(139.00,208.00) | 164.00(140.75,187.50) | .06 |
| POD7 BUN | 2.45(1.88,3.32) | 2.70(2.13,3.41) | 2.70(2.20,3.23) | .30 |
| POD7 Cr | 51.00(46.00,59.00) | 53.00(47.00,60.00) | 53.00(47.00,59.00) | .95 |
| POD7 UA | 190.95(145.25,230.33) | 169.15(139.55，210.00) | 172.60(151.00,203.00) | .27 |
| POD30 ALT | 16.00(12.00,29.00) | 16.00(12.00,23.00) | 17.00(13.00,23.00) | .96 |
| POD30 AST | 19.00(15.00,25.00) | 19.00(15.00,24.00) | 18.00(16.00,22.00) | .93 |
| POD30 TBil | 8.20(6.30,10.30) | 8.00(6.10,9.60) | 8.10(5.90,9.90) | .87 |
| POD30 ALP | 71.00(58.00,90.00) | 69.00(58.00,85.00) | 67.00(56.00,78.00) | .33 |
| POD30 γ-GTP | 32.00(19.25,51.50) | 29.00(20.00,52.00) | 24.50(19.00,35.00) | .08 |
| POD30 LDH | 139.50(128.00,178.50) | 182.00(135.00,202.00) | 155.00(141.25,172.75) | .06 |
| POD30 BUN | 3.53(2.90,4.18) | 3.50(2.90,4.67) | 3.40(2.90,4.53) | .67 |
| POD30 Cr | 53.00(47.25,59.50) | 50.00(45.00,60.00) | 53.00(47.00,61.00) | .27 |
| POD30 UA | 261.90(226.75,321.05) | 253.80(206.15,306.33) | 260.00(207.30,315.03) | .49 |
| POD60 ALT | 15.00(11.00,24.00) | 16.50(12.00,22.00) | 18.00(11.00,27.50) | .54 |
| POD60 AST | 17.50(16.00,24.25) | 19.00(15.00,22.25) | 19.00(15.00,26.00) | .73 |
| POD60 TBil | 8.75(6.23,10.53) | 7.80(6.70,10.73) | 8.00(6.35,9.75) | .68 |
| POD60 ALP | 72.00(59.00,84.00) | 74.50(60.75,87.25) | 65.00(57.00,76.50) | .14 |
| POD60γ-GTP | 23.00(16.00,37.75) | 25.00(18.50,46.00) | 22.00(18.00,36.75) | .51 |
| POD60 LDH | 149.00(132.50,180.50) | 169.00(138.50,182.50) | 151.00(135.75,180.50) | .59 |
| POD60 BUN | 3.53(2.63,4.54) | 3.77(3.10,4.56) | 3.58(2.96,4.31) | .45 |
| POD60 Cr | 54.00(48.25,59.00) | 51.00(46.00,57.50) | 55.00(50.00,63.00) | .09 |
| POD60 UA | 275.50(204.70,319.00) | 255.90(208.25,306.98) | 257.30(215.60,295.50) | .68 |
| POD90 ALT | 15.50(9.00,25.75) | 18.00(13.00,29.00) | 16.00(12.00,35.00) | .11 |
| POD90 AST | 19.00(14.00,25.00) | 20.00(17.50,25.00) | 21.00(16.00,28.00) | .19 |
| POD90 TBil | 8.40(6.10,10.650) | 8.90(6.70,12.05) | 8.45(6.75,11.43) | .28 |
| POD90 ALP | 63.00(53.00,78.50) | 73.00(58.00,88.50) | 66.00(53.75,77.00) | .04 |
| POD90 γ-GTP | 20.50(13.00,37.50) | 23.00(16.75,45.75) | 25.00(17.00,38.50) | .24 |
| POD90 LDH | 141.00(134.75,171.00) | 163.00(143.50,169.00) | 168.00(149.00,189.00) | .05 |
| POD90BUN | 3.90(3.10,5.10) | 3.85(2.94,4.76) | 4.28(3.28,5.09) | .35 |
| POD90 Cr | 53.00(48.00,60.00) | 51.00(46.50,58.00) | 56.00(48.50,66.50) | .05 |
| POD90 UA | 267.30(235.60,330.30) | 255.90(218.90,318.30) | 284.00(241.60,338.05) | .21 |

ALT= glutamic-pyruvic transaminase, AST=glutami-oxalacetic transaminase, TBil=total bilrubin, ALP=alkaline phosphatase, γ-GTP=γ-glutamyl transpeptidase, LDH= lactic dehydrogenase, BUN=blood urea nitrogen, Cr=creatinine, UA=uric acid

The red p-value refers to that the p-value is less than 0.05, which has statistical significance.

**Supplementary table 2b**

| p-value | Half-FLU vs.FLU | FLU vs. Arg | Half-FLU vs. Arg |
| --- | --- | --- | --- |
| POD7ALT | 0.0209 | <0.0001 | .06 |
| POD7AST | 0.0086 | <0.0001 | .02 |
| POD7γ-GTP | 0.4076 | 0.0081 | .03 |
| POD90ALP | 0.0399 | 0.0208 | .88 |

ALT= glutamic-pyruvic transaminase, AST=glutami-oxalacetic transaminase, γ-GTP=γ-glutamyl transpeptidase, Cr=creatinine, ALP=alkaline phosphatase

The red p-value refers to that the p-value is less than 0.05/3, which has statistical significance.
