# Supplementary material for: Efficiency and safety evaluation of prophylaxes for venous thrombosis after gynecological surgery
Source: Medicine (Baltimore). 2020 Jun 19;99(25):e20928. doi: 10.1097/MD.0000000000020928 (PMC7310966; doi:10.1097/MD.0000000000020928)
Supplement: Supplemental Digital Content [file medi-99-e20928-s004.docx]

**Supplementary Table 3.** Laboratory test results on different days compared by the results on the first day after operation in 3 groups.

**Supplementary Table 3a.** Half-FLU (n=100)

|  | POD1 | POD7  p-value | POD30  p-value | POD60  p-value | POD90  p-value |
| --- | --- | --- | --- | --- | --- |
| ALT | 1.50(8.00,17.00) | 14.00(9.00,32.00) | 16.00(12.00,29.00) | 15.00(11.00,23.00) | 15.50(9.00,25.50) |
|  |  | .0032 | .0004 | .0011 | .0075 |
| AST | 16.00(12.00,21.00) | 19.50(14.00,33.00) | 19.00(15.00,25.00) | 17.50(16.00,24.00) | 19.00(14.00,25.00) |
|  |  | .0054 | .0019 | .0088 | .0292 |
| TBil | 5.90(4.50,7.80) | 6.15(4.60,7.40) | 8.20(6.30,1.30) | 8.75(6.30,1.50) | 8.40(6.30,1.40) |
|  |  | .9596 | <.0001 | <.0001 | .0002 |
| ALP | 5.00(42.00,59.00) | 57.50(47.00,72.00) | 71.00(58.00,9.00) | 72.00(59.00,83.00) | 63.00(53.00,77.00) |
|  |  | .0009 | <.0001 | <.0001 | <.0001 |
| γ-GTP | 12.00(9.00,2.00) | 29.00(17.00,45.50) | 32.00(19.50,51.00) | 23.00(16.00,37.50) | 2.50(13.00,37.00) |
|  |  | <.0001 | <.0001 | <.0001 | <.0001 |
| LDH | 161.00(137.00,176.00) | 147.00(127.00,165.00) | 139.50(128.00,173.00) | 149.00(135.00,178.00) | 141.00(135.50,17.00) |
|  |  | .0416 | .0969 | .2379 | .1516 |
| BUN | 3.70(2.74,4.60) | 2.45(1.90,3.30) | 3.53(2.90,4.16) | 3.53(2.67,4.53) | 3.90(3.10,5.10) |
|  |  | <.0001 | .6303 | .5692 | .2626 |
| Cr | 55.00(47.00,62.00) | 51.00(46.00,59.00) | 53.00(47.50,59.00) | 54.00(47.50,59.00) | 53.00(47.00,59.00) |
|  |  | .0662 | .2637 | .2035 | .1771 |

**Supplementary Table 3b.** FLU (n=102)

|  | POD1 | POD7  p-value | POD30  p-value | POD60  p-value | POD90  p-value |
| --- | --- | --- | --- | --- | --- |
| ALT | 1.00(8.00,15.00) | 24.50(13.00,44.00) | 16.00(12.00,23.00) | 16.50(12.00,22.00) | 18.00(13.00,29.00) |
|  |  | <.0001 | <.0001 | <.0001 | <.0001 |
| AST | 17.00(13.00,21.00) | 27.00(18.00,55.00) | 19.00(15.00,24.00) | 19.00(15.00,22.00) | 2.00(18.00,25.00) |
|  |  | <.0001 | .003 | .0179 | .0001 |
| TBil | 5.60(4.10,8.00) | 5.90(4.70,7.80) | 8.00(6.10,9.60) | 7.80(6.70,1.70) | 8.90(6.80,12.00) |
|  |  | .3734 | <.0001 | <.0001 | <.0001 |
| ALP | 49.00(43.00,59.00) | 58.00(47.00,69.00) | 69.00(58.00,85.00) | 74.50(61.00,87.00) | 73.00(58.00,88.00) |
|  |  | .0011 | <.0001 | <.0001 | <.0001 |
| γ-GTP | 13.00(9.00,21.00) | 33.00(17.00,6.00) | 29.00(2.00,51.00) | 25.00(19.00,46.00) | 23.00(17.00,45.00) |
|  |  | <.0001 | <.0001 | <.0001 | <.0001 |
| LDH | 161.00(134.00,184.00) | 172.00(139.00,208.00) | 182.00(135.00,202.00) | 169.00(14.00,182.00) | 163.00(147.00,165.00) |
|  |  | .577 | .2413 | .7605 | .9056 |
| BUN | 3.90(3.08,5.10) | 2.70(2.14,3.35) | 3.50(2.90,4.67) | 3.77(3.10,4.54) | 3.85(2.96,4.72) |
|  |  | <.0001 | .2108 | .4684 | .3673 |
| Cr | 56.00(5.00,64.00) | 53.00(47.00,6.00) | 49.00(45.00,56.00) | 51.00(45.00,56.00) | 51.00(46.00,57.00) |
|  |  | .0158 | <.0001 | .0006 | .0031 |

**Supplementary Table 3c.** Arg (n=104)

|  | POD1 | POD7  p-value | POD30  p-value | POD60  p-value | POD90  p-value |
| --- | --- | --- | --- | --- | --- |
| ALT | 11.00(8.00,15.00) | 12.00(9.00,19.00) | 17.00(13.00,23.00) | 18.00(11.00,27.00) | 16.00(12.00,35.00) |
|  |  | .0986 | <.0001 | <.0001 | <.0001 |
| AST | 16.00(12.00,19.00) | 16.00(12.00,21.00) | 18.00(16.00,22.00) | 19.00(15.00,26.00) | 21.00(16.00,28.00) |
|  |  | .6559 | .0001 | <.0001 | <.0001 |
| TBil | 6.20(4.50,8.70) | 6.15(4.80,7.40) | 8.10(5.90,9.90) | 8.00(6.40,9.60) | 8.45(6.80,11.40) |
|  |  | .6367 | .0072 | .0021 | .0002 |
| ALP | 48.50(4.00,59.00) | 53.00(42.00,63.00) | 67.00(56.00,78.00) | 65.00(57.00,76.00) | 66.00(54.00,77.00) |
|  |  | .1001 | <.0001 | <.0001 | <.0001 |
| γ-GTP | 12.00(9.00,18.00) | 21.00(16.00,3.00) | 24.50(19.00,35.00) | 22.00(18.00,36.50) | 25.00(17.00,37.00) |
|  |  | <.0001 | <.0001 | <.0001 | <.0001 |
| LDH | 158.00(133.00,184.00) | 164.00(141.50,187.00) | 155.00(142.00,172.00) | 151.00(137.50,18.00) | 168.00(149.00,189.00) |
|  |  | .8017 | .9283 | .867 | .3549 |
| BUN | 3.61(2.90,4.70) | 2.70(2.20,3.20) | 3.40(2.90,4.50) | 3.58(2.96,4.30) | 4.28(3.30,5.08) |
|  |  | <.0001 | .4366 | .6779 | .078 |
| Cr | 57.00(5.00,65.00) | 53.00(47.00,59.00) | 53.00(47.00,61.00) | 54.00(49.00,63.00) | 55.00(47.00,64.00) |
|  |  | .0072 | .0179 | .1176 | .2622 |
