# Supplementary material for: Efficiency and safety evaluation of prophylaxes for venous thrombosis after gynecological surgery
Source: Medicine (Baltimore). 2020 Jun 19;99(25):e20928. doi: 10.1097/MD.0000000000020928 (PMC7310966; doi:10.1097/MD.0000000000020928)
Supplement: Supplemental Digital Content [file medi-99-e20928-s005.docx]

**Supplementary Table 4. Number of thrombosis events in the gynecologic malignancy by histological type and site of cancer.**

| Thrombosis(+)/Total | Cervix | Ovary | Uterus | Vaginal stump | Total |
| --- | --- | --- | --- | --- | --- |
| Squamous cell carcinoma | 29/119 | 0/0 | 0/0 | 0/2 | 29/121 |
| Adenocarcinoma | 2/25 | 6/31 | 0/19 | 0/0 | 8/75 |
| Sarcoma | 0/1 | 0/1 | 0/1 | 0/0 | 0/3 |
| Clear cell carcinoma | 0/0 | 0/1 | 0/1 | 0/0 | 0/2 |
| Small cell carcinoma | 0/2 | 0/0 | 0/0 | 0/0 | 0/2 |
| Adenosquamous cell carcinoma | 1/1 | 0/0 | 0/0 | 0/0 | 1/1 |
| Total | 32/148 | 6/33 | 0/21 | 0/2 | 38/204 |
