# Supplementary material for: Efficiency and safety evaluation of prophylaxes for venous thrombosis after gynecological surgery
Source: Medicine (Baltimore). 2020 Jun 19;99(25):e20928. doi: 10.1097/MD.0000000000020928 (PMC7310966; doi:10.1097/MD.0000000000020928)
Supplement: Supplemental Digital Content [file medi-99-e20928-s006.docx]

**Supplementary Table 5. Differences of clotting function results in separate groups.**

| Items | Half-FLU | FLU | Arg | P-value |
| --- | --- | --- | --- | --- |
| POD1APTT | 37.25(35.40,41.00) | 38.00(34.90,4.70) | 37.40(34.90,4.50) | .5614 |
| POD1DD | 1.85(1.29,3.18) | 2.04(1.21,3.23) | 1.95(1.18,2.89) | .8064 |
| POD1FIB | 3.37(2.82,3.77) | 3.35(2.88,3.92) | 3.19(2.85,3.82) | .9911 |
| POD1Hb | 105.66(103.12,108.20) | 105.45(102.46,108.45) | 105.42(102.63,108.20) | .9916 |
| POD1PLT | 161.50(127.00,222.00) | 186.00(136.00,223.00) | 18.00(146.00,224.00) | .4257 |
| POD1PT | 15.25(14.97,15.54) | 15.29(15.06,15.51) | 15.05(14.82,15.28) | .3421 |
| POD7APTT | 36.80(34.50,39.50) | 36.40(34.40,38.50) | 36.30(33.50,38.70) | .7155 |
| POD7DD | 1.90(1.31,3.28) | 2.08(1.34,3.28) | 2.96(2.31,3.91) | .0001 |
| POD7FIB | 4.71(3.99,5.48) | 4.91(4.25,5.41) | 4.58(4.15,5.50) | .8936 |
| POD7Hb | 104.76(102.13,107.40) | 103.30(10.45,106.15) | 105.13(102.32,107.95) | .6137 |
| POD7PLT | 244.61(226.89,262.34) | 257.58(24.63,274.52) | 254.46(237.50,271.43) | .5425 |
| POD7PT | 13.60(13.20,14.10) | 13.50(13.00,13.90) | 13.70(13.25,14.30) | .0506 |
| POD30APTT | 37.35(34.15,4.15) | 34.60(33.65,36.90) | 37.10(34.50,38.80) | .1692 |
| POD30DD | 1.45(.98,2.37) | 1.80(.75,3.06) | 1.39(1.17,2.35) | .8495 |
| POD30FIB | 3.98(3.20,4.53) | 3.74(3.30,4.26) | 3.58(3.14,4.49) | .8787 |
| POD30Hb | 108.82(105.85,111.79) | 111.32(108.61,114.04) | 111.56(108.91,114.21) | .3241 |
| POD30Plt | 196.00(145.00,265.00) | 204.00(162.00,266.00) | 224.00(174.00,28.00) | .0649 |
| POD30PT | 13.65(13.15,14.30) | 13.35(12.75,14.70) | 13.70(13.00,14.30) | .9171 |
| POD60APTT | 37.10(34.80,39.80) | 36.15(32.40,38.50) | 36.40(33.20,4.60) | .755 |
| POD60DD | .73(.38,1.11) | .99(.57,1.75) | 1.14(.57,1.45) | .2979 |
| POD60FIB | 3.45(2.76,4.11) | 4.29(3.13,5.84) | 3.56(2.88,4.30) | .2007 |
| POD60Hb | 104.00(95.00,114.00) | 109.00(101.00,116.00) | 107.00(97.00,117.00) | .576 |
| POD60Plt | 179.00(143.00,221.00) | 212.00(161.00,249.00) | 184.00(155.00,23.00) | .2295 |
| POD60PT | 13.70(13.05,14.35) | 13.95(13.40,14.30) | 13.85(13.40,14.80) | .6458 |
| POD90APTT | 38.00(36.00,4.10) | 36.10(35.30,38.30) | 35.95(33.85,37.85) | .1809 |
| POD90DD | .63(.32,1.22) | 1.04(.64,1.84) | .76(.48,1.32) | .334 |
| POD90FIB | 2.92(2.50,3.61) | 3.52(3.26,4.29) | 3.51(2.98,4.73) | .1886 |
| POD90Hb | 104.20(10.95,107.45) | 105.36(101.90,108.82) | 106.59(102.90,11.28) | .6409 |
| POD90Plt | 18.00(132.00,235.00) | 178.00(14.00,225.00) | 182.50(152.50,246.00) | .4469 |
| POD90PT | 13.40(13.10,13.70) | 13.40(13.10,13.90) | 13.25(12.80,13.75) | .6587 |

Hb=hemoglobin, PLT=platelet count, PT=prothrombin time, FIB=fibrinogen, APTT= activated partial thromboplastin time, D-D=D-dimer

The red p-value refers to that the p-value is less than .05, which has statistical significance.
