# Supplementary material for: Efficiency and safety evaluation of prophylaxes for venous thrombosis after gynecological surgery
Source: Medicine (Baltimore). 2020 Jun 19;99(25):e20928. doi: 10.1097/MD.0000000000020928 (PMC7310966; doi:10.1097/MD.0000000000020928)
Supplement: Supplemental Digital Content [file medi-99-e20928-s007.docx]

**Supplementary Table 6. Univariate analysis of demographic, preoperative and intra-operative characteristics of patients who had and did not have thrombosis.**

| Variables | No. of patients | | Rate of thrombosis (%) | p-value |
| --- | --- | --- | --- | --- |
|  | Thrombosis (-) | Thrombosis (+) |  |  |
| Age group |  |  |  | .0099 |
| ＜50 | 125 | 11 | 8.09 |  |
| ≥50 | 88 | 21 | 19.27 |  |
| BMI group |  |  |  | .4595 |
| ＜24 | 79 | 16 | 16.84 |  |
| ≥24 | 37 | 5 | 11.9 |  |
| Preoperative blood pressure |  |  |  |  |
| ＜120/80 | 89 | 14 | 13.59 | .9917 |
| ≥120/80 | 83 | 13 | 13.54 |  |
| ＜140/90 | 155 | 23 | 12.92 | .6611 |
| ≥140/90 | 17 | 4 | 19.05 |  |
| ＜160/100 | 167 | 26 | 13.47 | .5881 |
| ≥160/100 | 5 | 1 | 16.67 |  |
| ＜180/110 | 171 | 27 | 13.64 | 1.0000 |
| ≥180/110 | 1 | 0 | 0 |  |
| Site of cancer |  |  |  | .0848 |
| Cervix | 148 | 23 | 13.45 |  |
| Ovary | 33 | 8 | 19.51 |  |
| OtherPOD30 | 23 | 0 | 0 |  |
| Histological type |  |  |  | .3845 |
| Squamous cell carcinoma | 96 | 18 | 15.79 |  |
| Adenocarcinoma | 70 | 7 | 9.09 |  |
| OtherPOD60 | 9 | 1 | 10 |  |
| Clinical stage |  |  |  | .8971 |
| Ⅰ-Ⅱ | 144 | 22 | 13.25 |  |
| Ⅲ-Ⅳ | 8 | 2 | 20 |  |
| lymphadenectomy |  |  |  | .5111 |
| yes | 4 | 1 | 20 |  |
| no | 206 | 31 | 13.08 |  |
| Hospital stays |  |  |  | .8450 |
| ＜15 | 62 | 10 | 13.89 |  |
| ≥15 | 142 | 26 | 15.48 |  |

BMI=body mass index

OtherPOD30 include 22 cases were diagnosed as endometrial cancer and 2 cases were diagnosed as recurrent cancer at vaginal stump after operation.

OtherPOD60 include 4 cases whose histological type were sarcoma,3 cases were clear cell carcinoma ,2 cases were small cell carcinoma and 1 case were adenosquamous carcinoma.

The red p-value refers to that the p-value is less than .05, which has statistical significance.

The average age of menopause in Chinese is about 50, so we use 50 years old as the cut off value.
