# Supplementary material for: Efficiency and safety evaluation of prophylaxes for venous thrombosis after gynecological surgery
Source: Medicine (Baltimore). 2020 Jun 19;99(25):e20928. doi: 10.1097/MD.0000000000020928 (PMC7310966; doi:10.1097/MD.0000000000020928)
Supplement: Supplemental Digital Content [file medi-99-e20928-s008.docx]

**Supplementary Table 7. Analysis of the relationship between laboratory test results and postoperative thrombosis**

| Items | No. of patients | | rate of thrombosis | p-value |
| --- | --- | --- | --- | --- |
|  | Thrombosis (-) | Thrombosis (+) |  |  |
| Preoperative total cholesterol |  |  |  | 1.0000 |
| ≤5.20 | 177 | 27 | 13.24 |  |
| ＞5.20 | 32 | 5 | 13.51 |  |
| Preoperative triglyceride |  |  |  | .9397 |
| ≤1.70 | 81 | 14 | 14.74 |  |
| ＞1.70 | 18 | 4 | 18.18 |  |
| Preoperative Hb |  |  |  |  |
| ＜90 | 201 | 29 | 12.61 | .4247 |
| ≥90 | 9 | 3 | 25 |  |
| ＜115 | 137 | 13 | 8.67 | .0075 |
| ≥115 | 73 | 19 | 2.65 |  |
| Preoperative PLT |  |  |  |  |
| ≤350 | 201 | 29 | 12.61 | .4247 |
| ＞350 | 9 | 3 | 25 |  |
| Preoperative PT |  |  |  |  |
| ＜11.5 | 206 | 31 | 13.08 | .2503 |
| ≥11.5 | 1 | 1 | 50 |  |
| ≤14.5 | 198 | 27 | 12 | .0337 |
| ＞14.5 | 9 | 5 | 35.71 |  |
| Preoperative FIB |  |  |  | .5515 |
| ≤4 | 180 | 26 | 12.62 |  |
| ＞4 | 27 | 6 | 18.18 |  |
| Preoperative APTT |  |  |  | 1.0000 |
| ＜29 | 205 | 32 | 13.5 |  |
| ≥29 | 1 | 0 | 0 |  |
| Preoperative D-D |  |  |  |  |
| ≤.5 | 76 | 9 | 1.59 | .3664 |
| ＞.5 | 31 | 7 | 18.42 |  |
| ≤1.5 | 99 | 12 | 1.81 | .1110 |
| ＞1.5 | 9 | 4 | 3.77 |  |
| ≤3.0 | 103 | 13 | 11.21 | .1095 |
| ＞3.0 | 5 | 3 | 37.5 |  |
| POD1 Hb |  |  |  |  |
| ＜90 | 182 | 21 | 1.34 | .0550 |
| ≥90 | 21 | 7 | 25 |  |
| ＜115 | 53 | 4 | 7.02 | .1737 |
| ≥115 | 150 | 24 | 13.79 |  |
| POD1 PLT |  |  |  | .4871 |
| ≤350 | 201 | 28 | 12.23 |  |
| ＞350 | 4 | 1 | 20 |  |
| POD1 PT |  |  |  | .0664 |
| ≤14.5 | 51 | 3 | 5.56 |  |
| ＞14.5 | 110 | 20 | 15.38 |  |
| POD1 FIB |  |  |  | .2467 |
| ≤4.0 | 123 | 15 | 1.87 |  |
| ＞4.0 | 38 | 8 | 17.39 |  |
| POD1 APTT |  |  |  | 1.0000 |
| ＜29 | 159 | 23 | 12.64 |  |
| ≥29 | 2 | 0 | 0 |  |
| POD1 D-D |  |  |  |  |
| ≤.5 | 2 | 0 | 0 | 1.0000 |
| ＞.5 | 157 | 22 | 12.29 |  |
| ≤1.5 | 50 | 2 | 3.85 | .0299 |
| ＞1.5 | 109 | 20 | 15.5 |  |
| ≤3.0 | 121 | 12 | 9.02 | .0318 |
| ＞3.0 | 38 | 10 | 2.83 |  |
| POD7 Hb |  |  |  |  |
| ＜90 | 167 | 21 | 11.17 | .0308 |
| ≥90 | 13 | 6 | 31.58 |  |
| ＜115 | 37 | 2 | 5.13 | .1033 |
| ≥115 | 143 | 25 | 14.88 |  |
| POD7 PLT |  |  |  | .6782 |
| ≤350 | 155 | 22 | 12.43 |  |
| ＞350 | 24 | 5 | 17.24 |  |
| POD7 PT |  |  |  |  |
| ＜11.5 | 169 | 22 | 11.52 | 1.0000 |
| ≥11.5 | 1 | 0 | 0 |  |
| ≤14.5 | 157 | 20 | 11.3 | 1.0000 |
| ＞14.5 | 13 | 2 | 13.33 |  |
| POD7 FIB |  |  |  | 1.0000 |
| ≤4.0 | 36 | 5 | 12.2 |  |
| ＞4.0 | 134 | 17 | 11.26 |  |
| POD7 APTT |  |  |  | 1.0000 |
| ＜29 | 165 | 22 | 11.76 |  |
| ≥29 | 2 | 0 | 0 |  |
| POD7 D-D |  |  |  |  |
| ≤.5 | 1 | 0 | 0 | 1.0000 |
| ＞.5 | 161 | 19 | 1.56 |  |
| ≤1.5 | 42 | 0 | 0 | .0247 |
| ＞1.5 | 120 | 19 | 13.67 |  |
| ≤3.0 | 108 | 8 | 6.9 | .0348 |
| ＞3.0 | 54 | 11 | 16.92 |  |

Hb=hemoglobin, PLT=platelet count, PT=prothrombin time, FIB=fibrinogen, APTT= activated partial thromboplastin time, D-D=D-dimer

The red p-value refers to that the p-value is less than .05, which has statistical significance.
