# Supplementary material for: Efficiency and safety evaluation of prophylaxes for venous thrombosis after gynecological surgery
Source: Medicine (Baltimore). 2020 Jun 19;99(25):e20928. doi: 10.1097/MD.0000000000020928 (PMC7310966; doi:10.1097/MD.0000000000020928)
Supplement: Supplemental Digital Content [file medi-99-e20928-s009.docx]

**Supplementary Table 8. Univariate analysis of demographic, preoperative and intra-operative characteristics of patients who had and did not have thrombosis.**

**Half-FLU**

| Variables | Thrombosis (-)  N=70 | Thrombosis (+)  N=13 | p-value |
| --- | --- | --- | --- |
| Age (years) | 47.41(45.14,49.69) | 54.38(5.76,58.01) | .0133 |
| Hospital stays | 18(14,20) | 22(19,24) | .0275 |
| BMI | 23.49(22.33,24.65) | 22.08(2.16,24.01) | .2323 |
| Preoperative systolic pressure | 114(105,127) | 110(105,112) | .2274 |
| Preoperative diastolic pressure | 73.50(7.44,76.56) | 71.56(64.27,78.84) | .6248 |

BMI=body mass index

The item signed by “*” means that the data collected by this item do not satisfy normal distribution, so we use proc npar1way instead of T test, and their level are expressed by median (Quarterback spacing).

The red p-value refers to that the p-value is less than .05, which has statistical significance.

**FLU**

| Variables | Thrombosis (-)  N=73 | Thrombosis (+)  N=5 | p-value |
| --- | --- | --- | --- |
| Age (years) | 48.74(46.71,5.77) | 55.40(48.74,62.06) | .0967 |
| Hospital stays | 19(18,20) | 18(13,24) | .7175 |
| BMI | 22.31(21.30,23.32) | 22.38(16.65,28.11) | .9665 |
| Preoperative systolic pressure | 117(108,126) | 120(111,123) | .9804 |
| Preoperative diastolic pressure | 77.50(68.00,83.00) | 82.00(73.00,85.00) | .4621 |

BMI=body mass index

The item signed by “*” means that the data collected by this item do not satisfy normal distribution, so we use proc npar1way instead of T test, and their level are expressed by median (Quarterback spacing).

The red p-value refers to that the p-value is less than .05, which has statistical significance.

**Arg**

| Variables | Thrombosis (-)  N=70 | Thrombosis (+)  N=14 | p-value |
| --- | --- | --- | --- |
| Age (years) | 48.03(45.97,5.08) | 48.21(42.50,53.93) | .9430 |
| Hospital stays | 18(17,19) | 20(17,23) | .3032 |
| BMI | 23.02(21.96,24.08) | 2.94(18.02,23.87) | .1163 |
| Preoperative systolic pressure | 115(110,120) | 119(109,128) | .4905 |
| Preoperative diastolic pressure | 72.43(69.56,75.30) | 74.62(65.11,84.12) | .5515 |

BMI=body mass index
