# Supplementary material for: Efficiency and safety evaluation of prophylaxes for venous thrombosis after gynecological surgery
Source: Medicine (Baltimore). 2020 Jun 19;99(25):e20928. doi: 10.1097/MD.0000000000020928 (PMC7310966; doi:10.1097/MD.0000000000020928)
Supplement: Supplemental Digital Content [file medi-99-e20928-s010.docx]

**Supplementary Table 9. Univariate analysis of demographic, preoperative and intra-operative characteristics of patients who had and did not have thrombosis.**

**Half-FLU**

| Variables | No. of patients | | Rate of thrombosis (%) | p-value |
| --- | --- | --- | --- | --- |
|  | Thrombosis (-) | Thrombosis (+) |  |  |
| Age group |  |  |  | .0057 |
| ＜50 | 41 | 2 | 4.65 |  |
| ≥50 | 29 | 11 | 27.5 |  |
| BMI group |  |  |  | 1.0000 |
| ＜24 | 21 | 6 | 22.22 |  |
| ≥24 | 11 | 3 | 21.43 |  |
| Preoperative blood pressure |  |  |  |  |
| ＜120/80 | 30 | 6 | 16.67 | .7950 |
| ≥120/80 | 24 | 3 | 11.11 |  |
| ＜140/90 | 48 | 9 | 15.79 | .5811 |
| ≥140/90 | 6 | 0 | 0 |  |
| ＜160/100 | 53 | 9 | 14.52 | 1.0000 |
| ≥160/100 | 1 | 0 | 0 |  |
| ＜180/110 | 53 | 9 | 14.52 | 1.0000 |
| ≥180/110 | 1 | 0 | 0 |  |
| Site of cancer |  |  |  | .3007 |
| Cervix | 49 | 9 | 15.52 |  |
| Ovary | 11 | 4 | 26.67 |  |
| OtherPOD30 | 7 | 0 | 0 |  |
| Histological type |  |  |  | 1.0000 |
| Squamous cell carcinoma | 28 | 6 | 17.65 |  |
| Adenocarcinoma | 24 | 4 | 14.29 |  |
| OtherPOD60 | 4 | 0 | 0 |  |
| Clinical stage |  |  |  | .2797 |
| Ⅰ-Ⅱ | 50 | 8 | 13.79 |  |
| Ⅲ-Ⅳ | 1 | 1 | 50 |  |
| lymphadenectomy |  |  |  | 1.0000 |
| yes | 1 | 0 | 0 |  |
| no | 69 | 13 | 15.85 |  |
| Hospital stays |  |  |  | .8450 |
| ＜15 | 62 | 10 | 13.89 |  |
| ≥15 | 142 | 26 | 15.48 |  |

BMI=body mass index

OtherPOD30 include 22 cases were diagnosed as endometrial cancer and 2 cases were diagnosed as recurrent cancer at vaginal stump after operation.

OtherPOD60 include 4 cases whose histological type were sarcoma,3 cases were clear cell carcinoma ,2 cases were small cell carcinoma and 1 case were adenosquamous carcinoma.

The red p-value refers to that the p-value is less than .05, which has statistical significance.

The average age of menopause in Chinese is about 50, so we use 50 years old as the cut off value.

**FLU**

| Variables | No. of patients | | Rate of thrombosis (%) | p-value |
| --- | --- | --- | --- | --- |
|  | Thrombosis (-) | Thrombosis (+) |  |  |
| Age group |  |  |  | .0831 |
| ＜50 | 37 | 0 | 0 |  |
| ≥50 | 36 | 5 | 12.2 |  |
| BMI group |  |  |  | 1.0000 |
| ＜24 | 33 | 3 | 8.33 |  |
| ≥24 | 11 | 1 | 8.33 |  |
| Preoperative blood pressure |  |  |  |  |
| ＜120/80 | 27 | 1 | 3.57 | .5388 |
| ≥120/80 | 33 | 4 | 1.81 |  |
| ＜140/90 | 53 | 4 | 7.02 | .4931 |
| ≥140/90 | 7 | 1 | 12.5 |  |
| ＜160/100 | 58 | 5 | 7.94 | 1.0000 |
| ≥160/100 | 2 | 0 | 0 |  |
| ＜180/110 | 60 | 5 | 7.69 | - |
| ≥180/110 | 0 | 0 |  |  |
| Site of cancer |  |  |  | 1.0000 |
| Cervix | 52 | 4 | 7.14 |  |
| Ovary | 11 | 0 | 0 |  |
| OtherPOD30 | 7 | 0 | 0 |  |
| Histological type |  |  |  | .6949 |
| Squamous cell carcinoma | 36 | 4 | 10 |  |
| Adenocarcinoma | 23 | 1 | 4.17 |  |
| OtherPOD60 | 2 | 0 | 0 |  |
| Clinical stage |  |  |  | 1.0000 |
| Ⅰ-Ⅱ | 52 | 4 | 7.14 |  |
| Ⅲ-Ⅳ | 3 | 0 | 0 |  |
| lymphadenectomy |  |  |  | 1.0000 |
| yes | 2 | 0 | 0 |  |
| no | 71 | 5 | 6.58 |  |
| Hospital stays |  |  |  | .8450 |
| ＜15 | 62 | 10 | 13.89 |  |
| ≥15 | 142 | 26 | 15.48 |  |

BMI=body mass index

OtherPOD30 include 22 cases were diagnosed as endometrial cancer and 2 cases were diagnosed as recurrent cancer at vaginal stump after operation.

OtherPOD60 include 4 cases whose histological type were sarcoma,3 cases were clear cell carcinoma ,2 cases were small cell carcinoma and 1 case were adenosquamous carcinoma.

The red p-value refers to that the p-value is less than .05, which has statistical significance.

The average age of menopause in Chinese is about 50, so we use 50 years old as the cut off value.

**Arg**

| Variables | No. of patients | | Rate of thrombosis (%) | p-value |
| --- | --- | --- | --- | --- |
|  | Thrombosis (-) | Thrombosis (+) |  |  |
| Age group |  |  |  | 1.0000 |
| ＜50 | 47 | 9 | 16.07 |  |
| ≥50 | 23 | 5 | 17.86 |  |
| BMI group |  |  |  | .3378 |
| ＜24 | 25 | 7 | 21.88 |  |
| ≥24 | 15 | 1 | 6.25 |  |
| Preoperative blood pressure |  |  |  |  |
| ＜120/80 | 32 | 7 | 17.95 | .9308 |
| ≥120/80 | 26 | 6 | 18.75 |  |
| ＜140/90 | 54 | 10 | 15.63 | .2098 |
| ≥140/90 | 4 | 3 | 42.86 |  |
| ＜160/100 | 56 | 12 | 17.65 | .4601 |
| ≥160/100 | 2 | 1 | 33.33 |  |
| ＜180/110 | 58 | 13 | 18.31 | - |
| ≥180/110 | 0 | 0 | - |  |
| Site of cancer |  |  |  | .2599 |
| Cervix | 47 | 10 | 17.54 |  |
| Ovary | 11 | 4 | 26.67 |  |
| OtherPOD30 | 9 | 0 | 0 |  |
| Histological type |  |  |  | .2599 |
| Squamous cell carcinoma | 47 | 10 | 17.54 |  |
| Adenocarcinoma | 11 | 4 | 26.67 |  |
| OtherPOD60 | 9 | 0 | 0 |  |
| Clinical stage |  |  |  | 1.0000 |
| Ⅰ-Ⅱ | 42 | 10 | 19.23 |  |
| Ⅲ-Ⅳ | 4 | 1 | 20 |  |
| lymphadenectomy |  |  |  | .3176 |
| yes | 1 | 1 | 50 |  |
| no | 66 | 13 | 16.46 |  |
| Hospital stays |  |  |  | .8450 |
| ＜15 | 62 | 10 | 13.89 |  |
| ≥15 | 142 | 26 | 15.48 |  |

BMI=body mass index

OtherPOD30 include 22 cases were diagnosed as endometrial cancer and 2 cases were diagnosed as recurrent cancer at vaginal stump after operation.

OtherPOD60 include 4 cases whose histological type were sarcoma,3 cases were clear cell carcinoma ,2 cases were small cell carcinoma and 1 case were adenosquamous carcinoma.

The red p-value refers to that the p-value is less than .05, which has statistical significance.

The average age of menopause in Chinese is about 50, so we use 50 years old as the cut off value.
