# Supplementary material for: Efficiency and safety evaluation of prophylaxes for venous thrombosis after gynecological surgery
Source: Medicine (Baltimore). 2020 Jun 19;99(25):e20928. doi: 10.1097/MD.0000000000020928 (PMC7310966; doi:10.1097/MD.0000000000020928)
Supplement: Supplemental Digital Content [file medi-99-e20928-s011.docx]

**Supplementary Table 10. Analysis of the relationship between laboratory test results and postoperative thrombosis**

**Half-FLU**

| Items | Thrombosis (-) | Thrombosis (+) | p-value |
| --- | --- | --- | --- |
| preTC | 4.17(3.95,4.38) | 4.58(4.07,5.10) | .1285 |
| preTG | .94(.73,1.23) | 1.85(.89,2.30) | .0772 |
| preAPTT | 36.20(34.20,38.00) | 37.60(35.70,39.90) | .2361 |
| preDD | .32(.27,.50) | .34(.29,.45) | .7961 |
| preFIB | 2.99(2.60,3.53) | 3.39(2.40,4.34) | .6129 |
| preHb | 117.54(114.28,12.81) | 111.00(102.90,119.10) | .1123 |
| prePLT_ | 202.50(164.00,255.00) | 204.00(153.00,25.00) | .8424 |
| prePT | 13.40(13.00,13.70) | 13.80(13.50,14.60) | .0034 |
| POD1APTT | 36.80(35.20,4.80) | 39.40(36.90,41.60) | .1369 |
| POD1DD | 1.85(1.36,2.88) | 3.14(2.07,4.34) | .0399 |
| POD1FIB | 3.35(2.79,3.79) | 3.44(3.01,4.02) | .6446 |
| POD1Hb | 106.64(103.66,109.61) | 94.65(86.68,102.62) | .0024 |
| POD1PLT | 171.50(144.00,222.00) | 139.50(119.00,27.00) | .2958 |
| POD1PT | 15.17(14.83,15.51) | 15.54(14.28,16.81) | .4295 |
| POD7APTT | 36.81(35.67,37.94) | 36.32(34.48,38.16) | .7290 |
| POD7DD | 1.69(1.23,3.01) | 2.98(2.80,3.88) | .0060 |
| POD7FIB | 4.86(4.63,5.09) | 4.26(3.35,5.17) | .0647 |
| POD7Hb | 105.94(102.75,109.12) | 96.90(9.93,102.87) | .0306 |
| POD7PLT | 25.73(228.76,272.71) | 238.10(192.47,283.73) | .6556 |
| POD7PT | 13.59(13.41,13.78) | 13.86(13.58,14.14) | .2474 |

Hb=hemoglobin, PLT=platelet count, PT=prothrombin time, FIB=fibrinogen, APTT= activated partial thromboplastin time, D-D=D-dimer

The red p-value refers to that the p-value is less than .05, which has statistical significance.

FLU

| Items | Thrombosis (-) | Thrombosis (+) | p-value |
| --- | --- | --- | --- |
| preTC | 4.38(4.16,4.61) | 4.47(2.79,6.14) | .8543 |
| preTG | .98(.78,1.49) | 1.53(1.49,1.56) | .2358 |
| preAPTT | 36.96(36.02,37.90) | 35.14(32.25,38.03) | .3113 |
| preDD | .40(.27,.58) | 1.15(.45,5.94) | .0489 |
| preFIB | 3.08(2.57,3.79) | 3.14(2.87,3.80) | .6239 |
| preHb | 116.38(112.64,12.13) | 113.60(95.64,131.56) | .7071 |
| prePLT_ | 219.00(181.00,261.00) | 259.00(246.00,294.00) | .0798 |
| prePT | 13.59(13.41,13.78) | 13.58(12.63,14.53) | .9682 |
| POD1APTT | 37.30(34.80,4.70) | 39.80(34.00,47.60) | .5867 |
| POD1DD | 1.94(1.20,3.06) | 2.97(2.12,3.94) | .1922 |
| POD1FIB | 3.45(3.23,3.68) | 4.24(2.20,6.28) | .0775 |
| POD1Hb | 105.76(102.27,109.26) | 108.50(92.04,124.96) | .7185 |
| PLT | 187.00(138.00,223.50) | 20.00(161.50,234.50) | .6022 |
| POD1PT | 15.26(15.00,15.51) | 15.13(13.81,16.44) | .7837 |
| POD7APTT | 36.68(35.84,37.52) | 34.07(24.97,43.16) | .1709 |
| POD7DD | 2.08(1.31,3.34) | 3.99(2.75,5.23) | .2055 |
| POD7FIB | 4.82(4.54,5.09) | 5.02(3.98,6.07) | .7394 |
| POD7Hb | 103.40(10.61,106.19) | 96.66(72.01,121.31) | .4931 |
| POD7PLT | 259.75(237.81,281.69) | 302.60(233.42,371.78) | .2738 |
| POD7PT | 13.53(13.37,13.70) | 13.60(12.94,14.26) | .8572 |

Hb=hemoglobin, PLT=platelet count, PT=prothrombin time, FIB=fibrinogen, APTT= activated partial thromboplastin time, D-D=D-dimer

The red p-value refers to that the p-value is less than .05, which has statistical significance.

Arg

| Items | Thrombosis (-) | Thrombosis (+) | p-value |
| --- | --- | --- | --- |
| preTC | 4.33(4.11,4.56) | 4.20(3.86,4.55) | .6124 |
| preTG | 1.09(.80,1.82) | .99(.89,1.24) | .5127 |
| preAPTT | 36.73(35.89,37.56) | 35.36(33.77,36.96) | .1670 |
| preDD | .40(.30,.59) | .52(.31,1.14) | .3184 |
| preFIB | 3.04(2.73,3.52) | 3.26(2.79,3.76) | .5340 |
| preHb | 12.94(116.97,124.92) | 11.14(99.84,12.44) | .0309 |
| prePLT_ | 208.00(179.00,268.00) | 279.50(207.00,311.00) | .0515 |
| prePT | 13.27(13.12,13.42) | 13.38(12.76,13.99) | .7181 |
| POD1APTT | 37.90(34.60,4.50) | 36.45(33.60,36.90) | .0885 |
| POD1DD | 1.87(1.10,2.66) | 3.06(2.05,4.07) | .0235 |
| POD1FIB | 3.17(2.85,4.34) | 3.62(2.66,4.06) | .6068 |
| POD1Hb | 106.85(103.65,11.04) | 102.13(93.54,11.71) | .2506 |
| POD1PLT | 17.00(145.00,217.00) | 211.00(142.00,227.00) | .7063 |
| POD1PT | 14.83(14.55,15.10) | 15.86(15.25,16.47) | .0031 |
| POD7APTT | 36.66(35.49,37.84) | 34.80(31.98,37.62) | .2230 |
| POD7DD | 2.74(2.30,3.71) | 4.15(2.72,5.28) | .0812 |
| POD7FIB | 4.58(4.16,5.46) | 5.77(4.44,6.72) | .1594 |
| POD7Hb | 106.22(103.20,109.24) | 103.58(96.57,11.60) | .4718 |
| POD7PLT | 246.22(228.48,263.96) | 28.08(20.79,359.37) | .3788 |
| POD7PT | 13.65(13.41,13.88) | 14.27(13.79,14.74) | .0422 |

Hb=hemoglobin, PLT=platelet count, PT=prothrombin time, FIB=fibrinogen, APTT= activated partial thromboplastin time, D-D=D-dimer

The red p-value refers to that the p-value is less than .05, which has statistical significance.
