# Supplementary material for: Efficiency and safety evaluation of prophylaxes for venous thrombosis after gynecological surgery
Source: Medicine (Baltimore). 2020 Jun 19;99(25):e20928. doi: 10.1097/MD.0000000000020928 (PMC7310966; doi:10.1097/MD.0000000000020928)
Supplement: Supplemental Digital Content [file medi-99-e20928-s012.docx]

**Supplementary Table 11. Analysis of the relationship between laboratory test results and postoperative thrombosis**

**Half-FLU**

| Items | No. of patients | | rate of thrombosis | p-value |
| --- | --- | --- | --- | --- |
|  | Thrombosis (-) | Thrombosis (+) |  |  |
| Preoperative total cholesterol |  |  |  | .3981 |
| ≤5.20 | 62 | 10 | 13.89 |  |
| ＞5.20 | 7 | 3 | 30 |  |
| Preoperative triglyceride |  |  |  | .0173 |
| ≤1.70 | 33 | 4 | 1.81 |  |
| ＞1.70 | 3 | 4 | 57.14 |  |
| Preoperative Hb |  |  |  |  |
| ＜90 | 66 | 12 | 15.38 | .4126 |
| ≥90 | 2 | 1 | 33.33 |  |
| ＜115 | 44 | 5 | 1.2 | .0761 |
| ≥115 | 24 | 8 | 25 |  |
| Preoperative PLT |  |  |  | .4126 |
| ≤350 | 66 | 12 | 15.38 |  |
| ＞350 | 2 | 1 | 33.33 |  |
| Preoperative PT |  |  |  |  |
| ＜11.5 | 68 | 13 | 16.05 | 1.0000 |
| ≥11.5 | 1 | 0 | 0 |  |
| ≤14.5 | 67 | 9 | 11.84 | .0050 |
| ＞14.5 | 2 | 4 | 66.67 |  |
| Preoperative FIB |  |  |  | .1717 |
| ≤4 | 61 | 9 | 12.86 |  |
| ＞4 | 8 | 4 | 33.33 |  |
| Preoperative APTT |  |  |  | - |
| ＜29 | 69 | 13 | 15.85 |  |
| ≥29 | 0 | 0 | - |  |
| Preoperative D-D |  |  |  |  |
| ≤.5 | 31 | 4 | 11.43 | 1.0000 |
| ＞.5 | 11 | 1 | 8.33 |  |
| ≤1.5 | 38 | 4 | 9.52 | .4454 |
| ＞1.5 | 4 | 1 | 20 |  |
| ≤3.0 | 40 | 4 | 9.09 | .2920 |
| ＞3.0 | 2 | 1 | 33.33 |  |
| POD1 Hb |  |  |  |  |
| ＜90 | 60 | 8 | 11.76 | .0656 |
| ≥90 | 6 | 4 | 40 |  |
| ＜115 | 15 | 0 | 0 | .1500 |
| ≥115 | 51 | 12 | 19.05 |  |
| POD1 PLT |  |  |  | .2857 |
| ≤350 | 65 | 11 | 14.47 |  |
| ＞350 | 1 | 1 | 50 |  |
| POD1 PT |  |  |  | .8924 |
| ≤14.5 | 17 | 2 | 1.53 |  |
| ＞14.5 | 38 | 7 | 15.56 |  |
| POD1 FIB |  |  |  | .644 |
| ≤4.0 | 44 | 6 | 12 |  |
| ＞4.0 | 11 | 3 | 21.43 |  |
| POD1 APTT |  |  |  | 1.0000 |
| ＜29 | 54 | 9 | 14.29 |  |
| ≥29 | 1 | 0 | 0 |  |
| POD1 D-D |  |  |  |  |
| ≤.5 | 54 | 8 | 12.9 | - |
| ＞.5 | 0 | 0 | - |  |
| ≤1.5 | 16 | 1 | 5.88 | .5559 |
| ＞1.5 | 38 | 7 | 15.56 |  |
| ≤3.0 | 41 | 5 | 1.87 | .7061 |
| ＞3.0 | 13 | 3 | 18.75 |  |
| POD7 Hb |  |  |  |  |
| ＜90 | 57 | 8 | 12.31 | .1969 |
| ≥90 | 4 | 2 | 33.33 |  |
| ＜115 | 13 | 0 | 0 | .2403 |
| ≥115 | 48 | 10 | 17.24 |  |
| POD7 PLT |  |  |  | 1.0000 |
| ≤350 | 51 | 9 | 15 |  |
| ＞350 | 9 | 1 | 10 |  |
| POD7 PT |  |  |  |  |
| ＜11.5 | 58 | 10 | 14.71 | - |
| ≥11.5 | 0 | 0 | - |  |
| ≤14.5 | 52 | 10 | 16.13 | .5814 |
| ＞14.5 | 6 | 0 | 0 |  |
| POD7 FIB |  |  |  | .4291 |
| ≤4.0 | 13 | 4 | 23.53 |  |
| ＞4.0 | 45 | 6 | 11.76 |  |
| POD7 APTT |  |  |  | 1.0000 |
| ＜29 | 55 | 10 | 15.38 |  |
| ≥29 | 2 | 0 | 0 |  |
| POD7 D-D |  |  |  |  |
| ≤.5 | 1 | 0 | 0 | 1.0000 |
| ＞.5 | 55 | 9 | 14.06 |  |
| ≤1.5 | 21 | 0 | 0 | .0645 |
| ＞1.5 | 35 | 9 | 2.45 |  |
| ≤3.0 | 41 | 5 | 1.87 | .4925 |
| ＞3.0 | 15 | 4 | 21.05 |  |

Hb=hemoglobin, PLT=platelet count, PT=prothrombin time, FIB=fibrinogen, APTT= activated partial thromboplastin time, D-D=D-dimer

The red p-value refers to that the p-value is less than .05, which has statistical significance.

**FLU**

| Items | No. of patients | | rate of thrombosis | p-value |
| --- | --- | --- | --- | --- |
|  | Thrombosis (-) | Thrombosis (+) |  |  |
| Preoperative total cholesterol |  |  |  | 1.0000 |
| ≤5.20 | 57 | 4 | 6.56 |  |
| ＞5.20 | 15 | 1 | 6.25 |  |
| Preoperative triglyceride |  |  |  | 1.0000 |
| ≤1.70 | 26 | 2 | 7.14 |  |
| ＞1.70 | 6 | 0 | 0 |  |
| Preoperative Hb |  |  |  |  |
| ＜90 | 69 | 5 | 6.76 | 1.0000 |
| ≥90 | 4 | 0 | 0 |  |
| ＜115 | 45 | 3 | 6.25 | 1.0000 |
| ≥115 | 28 | 2 | 6.67 |  |
| Preoperative PLT |  |  |  | 1.0000 |
| ≤350 | 67 | 5 | 6.94 |  |
| ＞350 | 6 | 0 | 0 |  |
| Preoperative PT |  |  |  |  |
| ＜11.5 | 71 | 5 | 6.58 | - |
| ≥11.5 | 0 | 0 | - |  |
| ≤14.5 | 64 | 5 | 7.25 | 1.0000 |
| ＞14.5 | 7 | 0 | 0 |  |
| Preoperative FIB |  |  |  | .5529 |
| ≤4 | 61 | 4 | 6.15 |  |
| ＞4 | 10 | 1 | 9.09 |  |
| Preoperative APTT |  |  |  | 1.0000 |
| ＜29 | 69 | 5 | 6.76 |  |
| ≥29 | 1 | 0 | 0 |  |
| Preoperative D-D |  |  |  |  |
| ≤.5 | 22 | 1 | 4.35 | .2390 |
| ＞.5 | 9 | 2 | 18.18 |  |
| ≤1.5 | 30 | 2 | 6.25 | .2422 |
| ＞1.5 | 2 | 1 | 33.33 |  |
| ≤3.0 | 31 | 2 | 6.06 | .1664 |
| ＞3.0 | 1 | 1 | 50 |  |
| POD1 Hb |  |  |  |  |
| ＜90 | 62 | 4 | 6.06 | 1.0000 |
| ≥90 | 10 | 0 | 0 |  |
| ＜115 | 19 | 2 | 9.52 | .6502 |
| ≥115 | 53 | 2 | 3.64 |  |
| POD1 PLT |  |  |  | 1.0000 |
| ≤350 | 70 | 4 | 5.41 |  |
| ＞350 | 2 | 0 | 0 |  |
| POD1 PT |  |  |  | 1.0000 |
| ≤14.5 | 13 | 1 | 7.14 |  |
| ＞14.5 | 40 | 3 | 6.98 |  |
| POD1 FIB |  |  |  | .2502 |
| ≤4.0 | 41 | 2 | 4.65 |  |
| ＞4.0 | 12 | 2 | 14.29 |  |
| POD1 APTT |  |  |  | 1.0000 |
| ＜29 | 52 | 4 | 7.14 |  |
| ≥29 | 1 | 0 | 0 |  |
| POD1 D-D |  |  |  |  |
| ≤.5 | 1 | 0 | 0 | 1.0000 |
| ＞.5 | 51 | 4 | 7.27 |  |
| ≤1.5 | 15 | 0 | 0 | .5032 |
| ＞1.5 | 37 | 4 | 9.76 |  |
| ≤3.0 | 38 | 2 | 5 | .6817 |
| ＞3.0 | 14 | 2 | 12.5 |  |
| POD7 Hb |  |  |  |  |
| ＜90 | 55 | 3 | 5.17 | .0857 |
| ≥90 | 5 | 2 | 28.57 |  |
| ＜115 | 10 | 0 | 0 | 10000 |
| ≥115 | 50 | 5 | 9.09 |  |
| POD7 PLT |  |  |  | 1.0000 |
| ≤350 | 48 | 4 | 7.69 |  |
| ＞350 | 12 | 1 | 7.69 |  |
| POD7 PT |  |  |  |  |
| ＜11.5 | 57 | 3 | 5 | 1.0000 |
| ≥11.5 | 1 | 0 | 0 |  |
| ≤14.5 | 56 | 3 | 5.08 | 1.0000 |
| ＞14.5 | 2 | 0 | 0 |  |
| POD7 FIB |  |  |  | 1.0000 |
| ≤4.0 | 13 | 0 | 0 |  |
| ＞4.0 | 45 | 3 | 6.25 |  |
| POD7 APTT |  |  |  | - |
| ＜29 | 57 | 3 | 5 |  |
| ≥29 | 0 | 0 | - |  |
| POD7 D-D |  |  |  |  |
| ≤.5 | 53 | 2 | 3.64 | - |
| ＞.5 | 0 | 0 | - |  |
| ≤1.5 | 19 | 0 | 0 | .5394 |
| ＞1.5 | 34 | 2 | 5.56 |  |
| ≤3.0 | 37 | 1 | 2.63 | .5266 |
| ＞3.0 | 16 | 1 | 5.88 |  |

Hb=hemoglobin, PLT=platelet count, PT=prothrombin time, FIB=fibrinogen, APTT= activated partial thromboplastin time, D-D=D-dimer

The red p-value refers to that the p-value is less than .05, which has statistical significance.

**Arg**

| Items | No. of patients | | rate of thrombosis | p-value |
| --- | --- | --- | --- | --- |
|  | Thrombosis (-) | Thrombosis (+) |  |  |
| Preoperative total cholesterol |  |  |  | .7448 |
| ≤5.20 | 58 | 13 | 18.31 |  |
| ＞5.20 | 10 | 1 | 9.09 |  |
| Preoperative triglyceride |  |  |  | .2051 |
| ≤1.70 | 22 | 8 | 26.67 |  |
| ＞1.70 | 9 | 0 | 0 |  |
| Preoperative Hb |  |  |  |  |
| ＜90 | 66 | 12 | 15.38 | .1961 |
| ≥90 | 3 | 2 | 40 |  |
| ＜115 | 48 | 5 | 9.43 | .0162 |
| ≥115 | 21 | 9 | 30 |  |
| Preoperative PLT |  |  |  | .0723 |
| ≤350 | 68 | 12 | 15 |  |
| ＞350 | 1 | 2 | 66.67 |  |
| Preoperative PT |  |  |  |  |
| ＜11.5 | 67 | 13 | 16.25 | .1728 |
| ≥11.5 | 0 | 1 | 100 |  |
| ≤14.5 | 67 | 13 | 16.25 | .1728 |
| ＞14.5 | 0 | 1 | 100 |  |
| Preoperative FIB |  |  |  | .8383 |
| ≤4 | 58 | 13 | 18.31 |  |
| ＞4 | 9 | 1 | 10 |  |
| Preoperative APTT |  |  |  | - |
| ＜29 | 67 | 14 | 17.28 |  |
| ≥29 | 0 | 0 | - |  |
| Preoperative D-D |  |  |  |  |
| ≤.5 | 23 | 4 | 14.81 | .5981 |
| ＞.5 | 11 | 4 | 26.67 |  |
| ≤1.5 | 31 | 6 | 16.22 | .2368 |
| ＞1.5 | 3 | 2 | 40 |  |
| ≤3.0 | 32 | 7 | 17.95 | .4787 |
| ＞3.0 | 2 | 1 | 33.33 |  |
| POD1 Hb |  |  |  |  |
| ＜90 | 60 | 9 | 13.04 | .1969 |
| ≥90 | 5 | 3 | 37.5 |  |
| ＜115 | 19 | 2 | 9.52 | .5857 |
| ≥115 | 46 | 10 | 17.86 |  |
| POD1 PLT |  |  |  | 1 |
| ≤350 | 66 | 13 | 16.46 |  |
| ＞350 | 1 | 0 | 0 |  |
| POD1 PT |  |  |  | .0382 |
| ≤14.5 | 21 | 0 | 0 |  |
| ＞14.5 | 32 | 10 | 23.81 |  |
| POD1 FIB |  |  |  | 1.0000 |
| ≤4.0 | 38 | 7 | 15.56 |  |
| ＞4.0 | 15 | 3 | 16.67 |  |
| POD1 APTT |  |  |  | - |
| ＜29 | 53 | 10 | 15.87 |  |
| ≥29 | 0 | 0 | - |  |
| POD1 D-D |  |  |  |  |
| ≤.5 | 1 | 0 | 5 | 1.0000 |
| ＞.5 | 52 | 10 | 2.93 |  |
| ≤1.5 | 19 | 1 | 5 | .2149 |
| ＞1.5 | 34 | 9 | 2.93 |  |
| ≤3.0 | 42 | 5 | 1.64 | .1205 |
| ＞3.0 | 11 | 5 | 31.25 |  |
| POD7 Hb |  |  |  |  |
| ＜90 | 55 | 10 | 15.38 | .5801 |
| ≥90 | 4 | 2 | 33.33 |  |
| ＜115 | 14 | 2 | 12.5 | .8770 |
| ≥115 | 45 | 10 | 18.18 |  |
| POD7 PLT |  |  |  | .0907 |
| ≤350 | 56 | 9 | 13.85 |  |
| ＞350 | 3 | 3 | 50 |  |
| POD7 PT |  |  |  |  |
| ＜11.5 | 54 | 9 | 14.29 | - |
| ≥11.5 | 0 | 0 | - |  |
| ≤14.5 | 49 | 7 | 12.5 | .5668 |
| ＞14.5 | 5 | 2 | 28.57 |  |
| POD7 FIB |  |  |  | .946 |
| ≤4.0 | 10 | 1 | 9.09 |  |
| ＞4.0 | 44 | 8 | 15.38 |  |
| POD7 APTT |  |  |  | - |
| ＜29 | 53 | 9 | 14.52 |  |
| ≥29 | 0 | 0 | - |  |
| POD7 D-D |  |  |  |  |
| ≤.5 | 53 | 8 | 13.11 | - |
| ＞.5 | 0 | 0 | - |  |
| ≤1.5 | 2 | 0 | 0 | 1.0000 |
| ＞1.5 | 51 | 8 | 13.56 |  |
| ≤3.0 | 30 | 2 | 6.25 | .1975 |
| ＞3.0 | 23 | 6 | 2.69 |  |

Hb=hemoglobin, PLT=platelet count, PT=prothrombin time, FIB=fibrinogen, APTT= activated partial thromboplastin time, D-D=D-dimer

The red p-value refers to that the p-value is less than .05, which has statistical significance.
