# Supplementary material for: Efficiency and safety evaluation of prophylaxes for venous thrombosis after gynecological surgery
Source: Medicine (Baltimore). 2020 Jun 19;99(25):e20928. doi: 10.1097/MD.0000000000020928 (PMC7310966; doi:10.1097/MD.0000000000020928)
Supplement: Supplemental Digital Content [file medi-99-e20928-s013.docx]

**Supplementary Table 12.** The liver and renal function results in separate groups based on age.

1. Age≥50

| Items | Half-FLU | FLU | Arg | P-value |
| --- | --- | --- | --- | --- |
| POD1 ALT | 11.00(8.00,18.75) | 11.00(8.00,15.50) | 11.00(9.00,16.00) | .672 |
| POD1 AST | 17.00(12.00,21.00) | 17.00(13.50,22.50) | 17.00(13.00,19.00) | .637 |
| POD1 TBil | 5.60(4.60,7.80) | 6.50(4.05,8.05) | 7.5(5.50,9.90) | .041 |
| POD1 ALP | 52.00(46.00,63.00) | 52.00(46.00,64.00) | 55.00(47.00,64.00) | .656 |
| POD1 γ-GTP | 13.00(9.00,30.50) | 14.00(9.00,23.50) | 16.00(9.00,19.00) | .966 |
| POD1 LDH | 167.50(144.50,190.75) | 163.50(141.00,174.00) | 176.00(144.50,219.50) | .643 |
| POD1 BUN | 3.80(3.00,4.59) | 4.35(3.70,5.43) | 4.20(3.10,5.70) | .184 |
| POD1 Cr | 57.00(49.00,62.00) | 59.00(53.00,66.00) | 59.00(54.00,70.00) | .362 |
| POD1 UA | 163.00(120.40,226.00) | 164.00(129.00,198.60) | 170.00(126.00,242.25) | .813 |
| POD7 ALT | 21.00(11.00,30.00) | 26.00(15.25,46.75) | 13.00(10.50,20.00) | .006 |
| POD7 AST | 25.00(16.00,33.00) | 27.50(19.00,60.75) | 15.00(11.50,23.50) | .002 |
| POD7 TBil | 6.30(5.09,7.30) | 6.10(5.05,8.58) | 6.70(5.25,7.50) | .916 |
| POD7 ALP | 63.00(50.00,74.00) | 63.00(51.50,73.00) | 58.00(46.00,66.00) | .144 |
| POD7 γ-GTP | 37.00(20.00,57.00) | 37.00(21.00,70.00) | 22.00(16.00,29.00) | .007 |
| POD7 LDH | 141.00(135.50,155.00) | 176.00(149.50,216.00) | 171.00(153.00,186.00) | .052 |
| POD7 BUN | 2.67(2.17,4.04) | 2.90(2.29,3.84) | 2.80(2.32,3.36) | .895 |
| POD7 Cr | 53.00(46.50,61.00) | 57.00(48.00,63.00) | 53.00(47.00,60.00) | .675 |
| POD7 UA | 216.10(159.60,234.90) | 171.50(140.00,211.23) | 177.00(149.90,203.50) | .052 |
| POD30 ALT | 20.00(12.00,32.00) | 17.50(14.00,23.00) | 16.00(11.75,20.00) | .478 |
| POD30 AST | 22.00(16.00,28.00) | 18.00(15.00,24.75) | 18.00(15.00,21.00) | .323 |
| POD30 TBil | 8.90(7.1,11.00) | 8.05(7.43,9.83) | 8.05(6.53,10.10) | .654 |
| POD30 ALP | 77.00(63.00,91) | 73.00(64.00,89.50) | 68.00(61.50,78.75) | .189 |
| POD30 γ-GTP | 38.00(28.75,60.75) | 36.00(22.00,64.00) | 22.50(19.00,30.75) | .001 |
| POD30 LDH | 146.00(132.25,188.25) | 175.50(145.25,198.00) | 161.00(148.00,200.00) | .383 |
| POD30 BUN | 3.69(2.96,4.43) | 3.87(3.31,4.88) | 3.51(3.05,4.49) | .234 |
| POD30 Cr | 54.00(49.50,63.25) | 52.00(47.00,60.00) | 50.00(47.00,64.00) | .352 |
| POD30 UA | 256.00(218.50,313.250) | 257.00(193.00,304.00) | 288.45(213.50,326.95) | .452 |
| POD60 ALT | 15.00(10.00,22.25) | 16.50(12.75,21.50) | 16.00(11.50,21.00) | .717 |
| POD60 AST | 19.00(16.00,25.25) | 20.00(16.00,22.00) | 17.00(15.00,22.00) | .246 |
| POD60 TBil | 8.85(5.675,10.125) | 10.10(7.10,12.20) | 7.60(6.40,8.75) | .080 |
| POD60 ALP | 72.00(59.75,81.50) | 75.00(62.00,83.00) | 68.00(57.50,79.50) | .422 |
| POD60γ-GTP | 22.50(17.00,37.75) | 32.00(17.50,69.00) | 21.00(17.50,30.50) | .216 |
| POD60 LDH | 153.00(142.00,193.75) | 150.50(137.75,167.00) | 147.00(130.00,177.00) | .274 |
| POD60 BUN | 4.00(2.97,4.83) | 4.23(3.56,4.75) | 3.81(2.87,4.40) | .329 |
| POD60 Cr | 55.00(51.75,64.25) | 54.00(48.00,57.50) | 54.00(49.50,65.00) | .149 |
| POD60 UA | 282.20(246.73,322.13) | 255.80(212.00,291.00) | 273.00(253.00,301.10) | .189 |
| POD90 ALT | 15.00(9.00,30.50) | 19.00(15.25,26.50) | 15.50(10.50,21.25) | .220 |
| POD90 AST | 20.00(14.00,28.00) | 22.00(19.25,24.00) | 20.00(16.00,23.75) | .523 |
| POD90 TBil | 8.70(5.58,15.28) | 11.10(8.53,13.23) | 7.60(6.60,9.80) | .132 |
| POD90 ALP | 62.50(54.50,88.25) | 72.50(64.75,83.75) | 68.00(54.00,83.00) | .544 |
| POD90 γ-GTP | 24.00(14.00,44.50) | 30.50(18.25,45.75) | 25.00(16.00,41.00) | .950 |
| POD90 LDH | 140.50(132.75,179.75) | 165.00(139.00,175.00) | 174.50(154.50,207.50) | .264 |
| POD90BUN | 4.40(3.28,5.60) | 3.95(3.12,4.59) | 4.10(2.88,5.33) | .599 |
| POD90 Cr | 57.00(52.00,67.50) | 54.00(49.50,56.00) | 60.50(48.00,68.50) | .121 |
| POD90 UA | 284.00(258.60,341.50) | 239.00(216.50,318.90) | 272.50(220.98,340.70) | .220 |

ALT= glutamic-pyruvic transaminase, AST=glutami-oxalacetic transaminase, TBil=total bilrubin, ALP=alkaline phosphatase, γ-GTP=γ-glutamyl transpeptidase, LDH= lactic dehydrogenase, BUN=blood urea nitrogen, Cr=creatinine, UA=uric acid

The red p-value refers to that the p-value is less than 0.05, which has statistical significance.

1. Age<50

| Items | Half-FLU | FLU | Arg | P-value |
| --- | --- | --- | --- | --- |
| POD1 ALT | 9.00(8.00,15.25) | 10.00(7.00,14.75) | 10.50(7.00,14.00) | .589 |
| POD1 AST | 15.00(12.00,20.75) | 15.50(12.75,18.50) | 15.00(12.00,18.00) | .958 |
| POD1 TBil | 6.65(4.58,7.93) | 5.30(4.07,7.65) | 5.70(4.09,8.10) | .311 |
| POD1 ALP | 44.50(39.00,54.50) | 47.00(38.00,55.25) | 44.00(37.00,54.00) | .839 |
| POD1 γ-GTP | 11.50(8.25,15.00) | 13.00(8.50,18.00) | 12.00(9.00,17.00) | .735 |
| POD1 LDH | 159.50(133.25,182.25) | 160.50(131.75,189.75) | 148.00(130.50,177.00) | .652 |
| POD1 BUN | 3.44(2.66,4.28) | 3.40(2.90,4.30) | 3.46(2.80,4.09) | .919 |
| POD1 Cr | 55.00(47.00,4.60) | 56.00(50.00,64.00) | 57.00(50.00,65.00) | .357 |
| POD1 UA | 158.00(124.00,203.20) | 160.00(122.00,207.68) | 176.10(132.00,207.00) | .882 |
| POD7 ALT | 11.00(9.00,40.00) | 21.50(11.50,43.25) | 11.00(7.50,19.00) | .009 |
| POD7 AST | 16.00(12.00,38.50) | 27.00(16.00,46.00) | 16.00(12.50,20.50) | .004 |
| POD7 TBil | 5.95(4.17,8.15) | 5.70(4.59,7.50) | 5.80(4.65,7.65) | .848 |
| POD7 ALP | 53.00(43.00,65.25) | 53.00(43.00,64.00) | 50.00(42.00,61.50) | .665 |
| POD7 γ-GTP | 22.00(13.00,38.00) | 25.00(15.00,57.00) | 18.50(14.00,35.75) | .573 |
| POD7 LDH | 152.50(114.25,173.50) | 157.00(132.00,228.00) | 157.00(121.00,191.00) | .400 |
| POD7 BUN | 2.11(1.75,2.81) | 2.62(2.00,3.14) | 2.68(1.80,3.16) | .192 |
| POD7 Cr | 49.00(44.00,58.00) | 50.50(44.75,57.25) | 53.00(47.25,56.75) | .354 |
| POD7 UA | 163.00(141.20,215.00) | 169.15(137.03,206.90) | 171.00(151.98,203.25) | .900 |
| POD30 ALT | 16.00(9.00,28.00) | 14.00(9.00,23.00) | 17.00(13.50,27.00) | .367 |
| POD30 AST | 17.00(15.00,25.00) | 19.00(15.00,23.00) | 19.00(16.00,23.00) | .675 |
| POD30 TBil | 7.10(5.80,9.50) | 7.70(5.50,9.60) | 8.10(5.15,9.70) | .977 |
| POD30 ALP | 65.00(49.00,84.00) | 67.00(55.00,81.00) | 64.00(56.00,77.00) | .834 |
| POD30 γ-GTP | 23.00(16.00,37.00) | 26.00(17.50,41.50) | 27.50(18.25,38.00) | .825 |
| POD30 LDH | 139.00(126.00,174.00) | 198.00(132.00,228.00) | 151.00(137.00,172.00) | .163 |
| POD30 BUN | 3.40(2.90,4.11) | 3.25(2.70,4.10) | 3.28(2.82,4.55) | .804 |
| POD30 Cr | 51.00(45.50,55.5) | 48.00(45.00,60.00) | 55.00(47.25,59.50) | .198 |
| POD30 UA | 272.20(235.85,322.35) | 251.60(207.60,309.00) | 254.70(204.10,306.00) | .379 |
| POD60 ALT | 16.00(11.00,25.00) | 16.50(10.00,23.25) | 19.50(11.00,36.75) | .285 |
| POD60 AST | 16.00(15.25,23.50) | 17.00(14.00,23.00) | 20.50(16.00,30.00) | .111 |
| POD60 TBil | 7.30(6.35,10.90) | 7.50(6.30,9.00) | 8.15(6.08,10.48) | .621 |
| POD60 ALP | 69.50(53.75,95.25) | 74.00(55.00,89.00) | 64.00(57.00,75.50) | .460 |
| POD60γ-GTP | 23.00(15.00,41.00) | 23.00(18.50,34.25) | 25.00(18.00,37.00) | .707 |
| POD60 LDH | 142.50(133.00,172.00) | 156.50(144.50,173.00) | 164.00(148.00,185.00) | .104 |
| POD60 BUN | 3.03(2.50,3.91) | 3.3(2.79,3.95) | 3.46(2.96,4.23) | .358 |
| POD60 Cr | 49.00(42.50,55.50) | 50.50(45.00,58.25) | 56.00(50.00,63.00) | .012 |
| POD60 UA | 239.10(199.20,308.65) | 256.00(200.75,326.35) | 236.15(211.50,294.63) | .716 |
| POD90 ALT | 16.00(9.00,23.00) | 14.00(11.50,34.50) | 22.00(12.00,40.00) | .051 |
| POD90 AST | 18.00(14.00,21.00) | 19.00(15.50,25.00) | 22.00(16.00,35.00) | .097 |
| POD90 TBil | 8.30(7.00,9.30) | 8.70(6.40,10.97) | 9.20(6.80,11.50) | .491 |
| POD90 ALP | 63.00(53.00,77.00) | 76.00(57.50,93.50) | 64.00(52.00,74.00) | .085 |
| POD90 γ-GTP | 17.00(13.00,31.00) | 23.00(16.25,53.5) | 25.00(17.00,36.25) | .114 |
| POD90 LDH | 142.50(133.00,172.00) | 156.50(144.50,173.00) | 164.00(148.00,185.00) | .230 |
| POD90BUN | 3.61(2.85,4.58) | 3.65(2.83,4.82) | 4.38(3.30,5.00) | .217 |
| POD90 Cr | 51.50(42.75,59.00) | 50.00(43.50,59.75) | 56.00(50.00,65.00) | .045 |
| POD90 UA | 246.15(227.52,328.78) | 264.90(224.25,320.35) | 297.50(243.00,341.50) | .307 |

ALT= glutamic-pyruvic transaminase, AST=glutami-oxalacetic transaminase, TBil=total bilrubin, ALP=alkaline phosphatase, γ-GTP=γ-glutamyl transpeptidase, LDH= lactic dehydrogenase, BUN=blood urea nitrogen, Cr=creatinine, UA=uric acid

The red p-value refers to that the p-value is less than 0.05, which has statistical significance.
